# The bears from Dmanisi and the first dispersal of early *Homo* out of Africa

**DOI:** 10.1038/s41598-019-54138-6

**Published:** 2019-11-28

**Authors:** Tsegai Medin, Bienvenido Martínez-Navarro, Joan Madurell-Malapeira, Borja Figueirido, Giorgi Kopaliani, Florent Rivals, Gocha Kiladze, Paul Palmqvist, David Lordkipanidze

**Affiliations:** 1 IPHES, Institut Català de Paleoecologia Humana i Evolució Social, Zona Educacional, 4, Campus Sescelades URV (Edifici W3), 43007 Tarragona, Spain; 2 Commission of Culture and Sports, Po.Box. 1500, Asmara, Eritrea; 3 ICREA, Pg. Lluís Companys 23, 08010 Barcelona, Spain; 4 Àrea de Prehistòria, Universitat Rovira i Virgili (URV), Avda. Catalunya 35, 43002 Tarragona, Spain; 5 Institut Català de Paleontologia Miquel Crusafont, Universitat Autònoma de Barcelona, Edifici ICTA-ICP, C/de les columnes s/n Campus de la UAB, Cerdanyola del Vallès, 08193 Barcelona, Spain; 6 Departamento de Ecología y Geología, Facultad de Ciencias, Universidad de Málaga, Campus de Teatinos, 29071 Málaga, Spain; 7 National Museum of Georgia, 0105 Tbilisi, Georgia; 8 Tbilisi State University, Tbilisi, Georgia

**Keywords:** Animal behaviour, Palaeoecology

## Abstract

We report on the taxonomy and paleodiet of the bear population that inhabited the emblematic palaeoanthropological Early Pleistocene (1.8 Ma) site of Dmanisi (Georgia), based on a dual approach combining morphometrics and microwear of upper and lower teeth. Given that the teeth of *Ursus etruscus* Cuvier, 1823 from Dmanisi show considerable size variability, their systematic position has been debated. However, a comparative study of the coefficients of variation for tooth size measurements in several modern bear species shows that the variability in tooth size of the ursid population from Dmanisi could result from sexual dimorphism. The analysis of tooth microwear indicates that these bears inhabited a mixed environment of open plain with forest patches, where they had a browsing diet with a substantial contribution of meat and/or fish. Comparative tooth morphometric analyses of modern ursids and fossil *U. etruscus* indicate that this extinct species had an omnivorous behavior similar to that of extant brown bears. The ecological interactions of the Dmanisi bears with other members of the large mammals community, including the first hominins that dispersed out of Africa, are discussed in the light of this new evidence.

## Introduction

Dmanisi, an Early Pleistocene site of Georgia located in the Mashavera river valley in the Lesser Caucasus (85 km southwest of Tibilisi; 44°210E, 41°90N) and dated at 1.85-1.77 Ma^1–5^, is a key palaeoanthropological site to understand the first dispersal of early *Homo* out of Africa (Fig. S1).

The temporal and geographic setting of Dmanisi, the preservational completeness of its faunal and hominin remains, and the huge record of Oldowan tools unearthed at this site open the possibility of setting up potential relationships of early *Homo* with other species of the Early Pleistocene large mammal fauna^6–9^ that inhabited this ecological scenario^10^, particularly the members of the carnivore guild.

The bone assemblage of Dmanisi preserves tooth and skeletal remains of 28 species of large mammals (See Supplementary Material), which represent a mix of species that evolved in Europe during the early-middle Villafranchian (e.g., elephant *Mammuthus meridionalis*) together with others that originated in Asia (mainly bovids) or in Africa (e.g., some carnivores like *Megantereon whitei* or *Pachycrocuta* sp.) but dispersed to Europe in the late Villafranchian^6–8,11,12^ (see also Supp. Table S1 in ref. ^4^). This assemblage includes several hypercarnivores such as the giant, short-faced hyena *Pachycrocuta* sp., the giant cheetah *Acinonyx pardinensis*, the saber-tooth cats *Homotherium latidens* and *M. whitei*, and the lynx *Lynx issiodorensis*, as well as a number of meso- and hypocarnivores like the bear *Ursus etruscus* and the canids *Canis etruscus* and *Vulpes alopecoides*^4,10^.

Much has been debated concerning the ecological interactions and competition for scavengeable resources between early *Homo* and the ossifragous *Pachycrocuta brevirostris*^9–11,13^, or between early *Homo* and the flesh-eating *M. whitei* and *Homotherium latidens*^6,14^. However, although disentangling the diet of *U. etruscus* preserved at Dmanisi could provide keys to a deeper understanding of the ecological scenario of the first human dispersal out of Africa, including the competition with other species that shared the omnivorous habits of early *Homo*, the ecological interactions between bears and hominins at this site are still unexplored.

In this paper, we (i) clarify the systematic position of the *Ursus* specimens preserved at Dmanisi; (ii) perform morphometric and microwear analysis of the upper and lower teeth to elucidate the potential feeding behaviour of this bear; and (iii) address other important palaeobiological aspects, such as the degree of sexual dimorphism. Finally, we discuss the ecological scenario of the first human dispersal out of Africa in the light of the new evidence on the overlap of the omnivorous niche among bears, pigs and hominins.

## The Fossil Record of Bears in Dmanisi

Based on the huge size differences, Vekua^15^ reported the presence of two different ursid species in the site of Dmanisi, *U. etruscus* for the large size specimens, and *Ursus* sp. for a few specimens of smaller size [two palate fragments (D255, D622), three mandibular fragments (D36, D218, and D1029), one p4 (D97), one m2 (D1253), one M1 (D623), one P4 (D626), and one McII (D209)]. However, Baryšnikov^16^, based on the morphology of the lower carnassial tooth (m1), unified them in a single taxon, creating the new subspecies *U. etruscus vekuai*. The latter taxonomy was followed by Wagner^17^.

Studies on tooth microwear in carnivorous mammals^18–22^, including bears^23–25^, provide clues on their feeding behavior. Münzel^26^ developed a combination of tooth microwear and isotopic analyses for studying Late Pleistocene and extant European bears. Relevant information on *U. etruscus* has been reported from several Villafranchian sites in Europe, including St. Vallier^27^ and Senèze in France^28,29^, Orce (i.e., Venta Micena, Fuente Nueva-3 and Barranco León-sites) in Spain^30,31^, Tegelen in the Netherlands^32^, Kuruksay inTadjikistan^33^, as well as Pirro Nord and Upper Valdarno in Italy^34,35^. Moreover, ursids from the site of Ubeidiya, in the Levantine corridor, fall within the variability of *U. etruscus*^12,36^. However, up to date, there are no studies on the tooth microwear patterns of the bears from Dmanisi.

## Results

### Systematic paleontology

Order Carnivora, Bowdich, 1821

Suborder Fissipeda, Simpson, 1945

Family Ursidae Gray, 1825

Genus *Ursus* Linnaeus, 1758

Species *Ursus etruscus* Cuvier, 1823

Referred specimens: See Supplementary Data.

### Anatomical description

#### Upper dentition

Few sexually dimorphic upper canines, P4, M1 and M2 were ascribed to Ursids in the Dmanisi collection. The three anterior premolars (P1-P3) are preserved, however they are strongly reduced in size compared to the proportions of other check teeth, as happens in *U. etruscus*.

**Figure 1:**
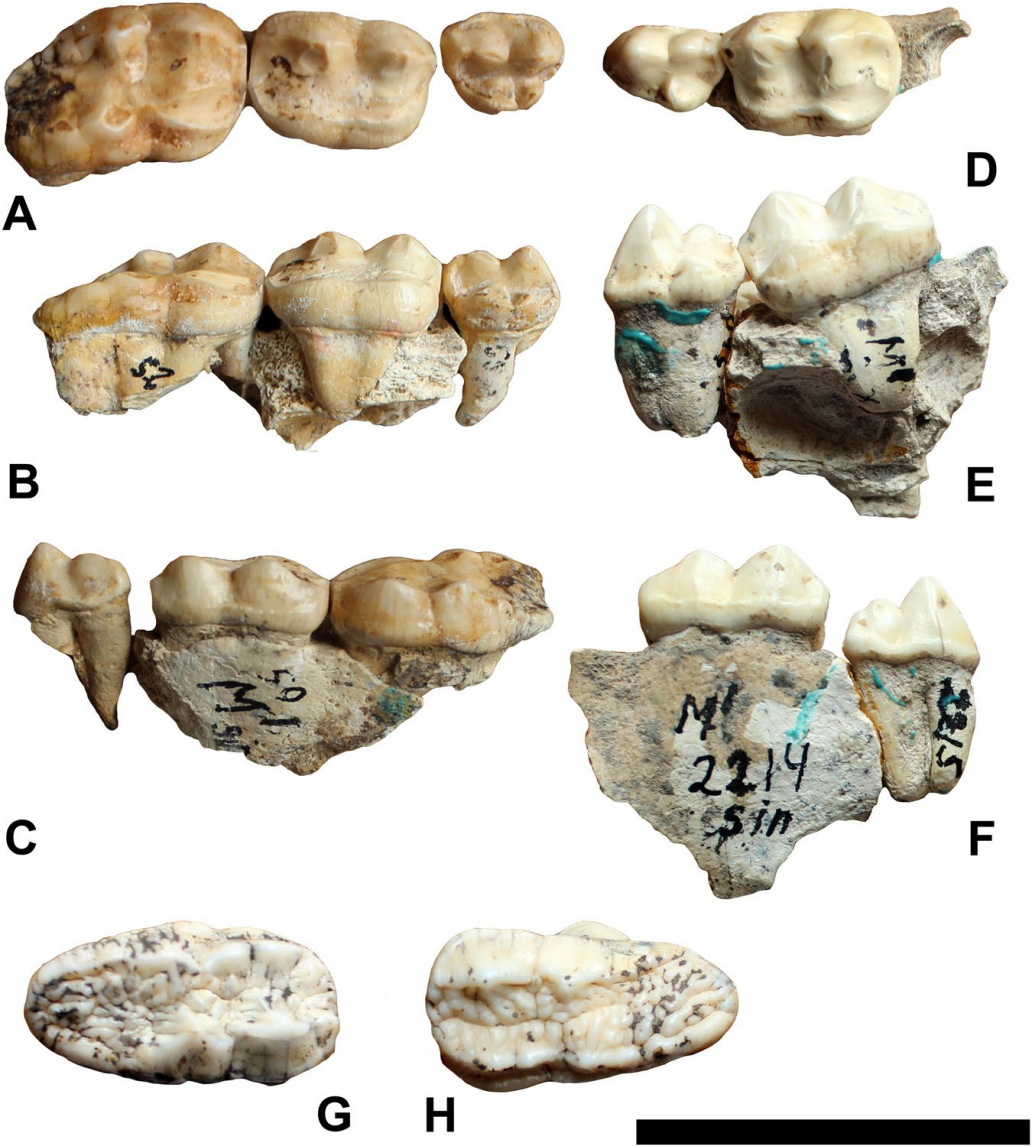
*Ursus etruscus* upper dental series. D50-52-55 in (**A**) occlusal view, (**B**) lingual view, (**C**) buccal view.D2214-2215 in (**D**) occlusal view, (**E**) lingual view, (**F**) buccal view. D4713 in (**G**) occlusal view. D2533 in (**H**) occlusal view. Scale bar = 5 cm.

The upper carnassial (P4) is buccolingually reduced in size, showing in the specimen D55 a sharply unicuspid paracone and metacone (Fig. 1A–F). The paracone is vertically oriented and the metacone is composed of a well-marked and individualized small cusp in the distobuccal region of the tooth. A low and individualized deuterocone is visible on the lingual side. Several specimens (e.g., D53) show a bicuspid deuterocone, with the smallest cuspid placed distolingually. In specimen D2215, the deuterocone is found at a lower position, with a single cusp and a crown that is slightly higher than in D55. A low depressed area separates the paracone from the metacone. A ridge marks the sides of the cingulum, mainly on the buccal side of the premolar.

The upper first molars (M1) have rounded corners (D4473, D50 and D2214; Fig. 1A–F), with a thick cingulum. A clear ridge is visible at the buccal side of the cingulum (D50). Their distal lobe is mesiodistally longer and buccolingually wider than the mesial one. The paracone and metacone are well marked, showing sharp and vertical cusps; both are comparable in size, showing a longitudinal crest and a conical shape. The parastyle and metastyle are both present, although they are small in size. The protocone and hypocone are both lowered relative to other dental cusps.

The upper second molars (M2) have a distally elongated talon, with a sharp and prominent paracone, a bicuspid metacone, a protocone and a bicuspid hypocone (D4713, D2533 and D52; Fig. 1A–C,G,H). The parastyle is visible at the mesial face of the paracone. There are four roots. The talon surface has a low heel and gently slopes distally to the distobuccal side. The buccal cusps of the occlusal surface are higher than the lingual ones.

#### Lower dentition

**Figure 2:**
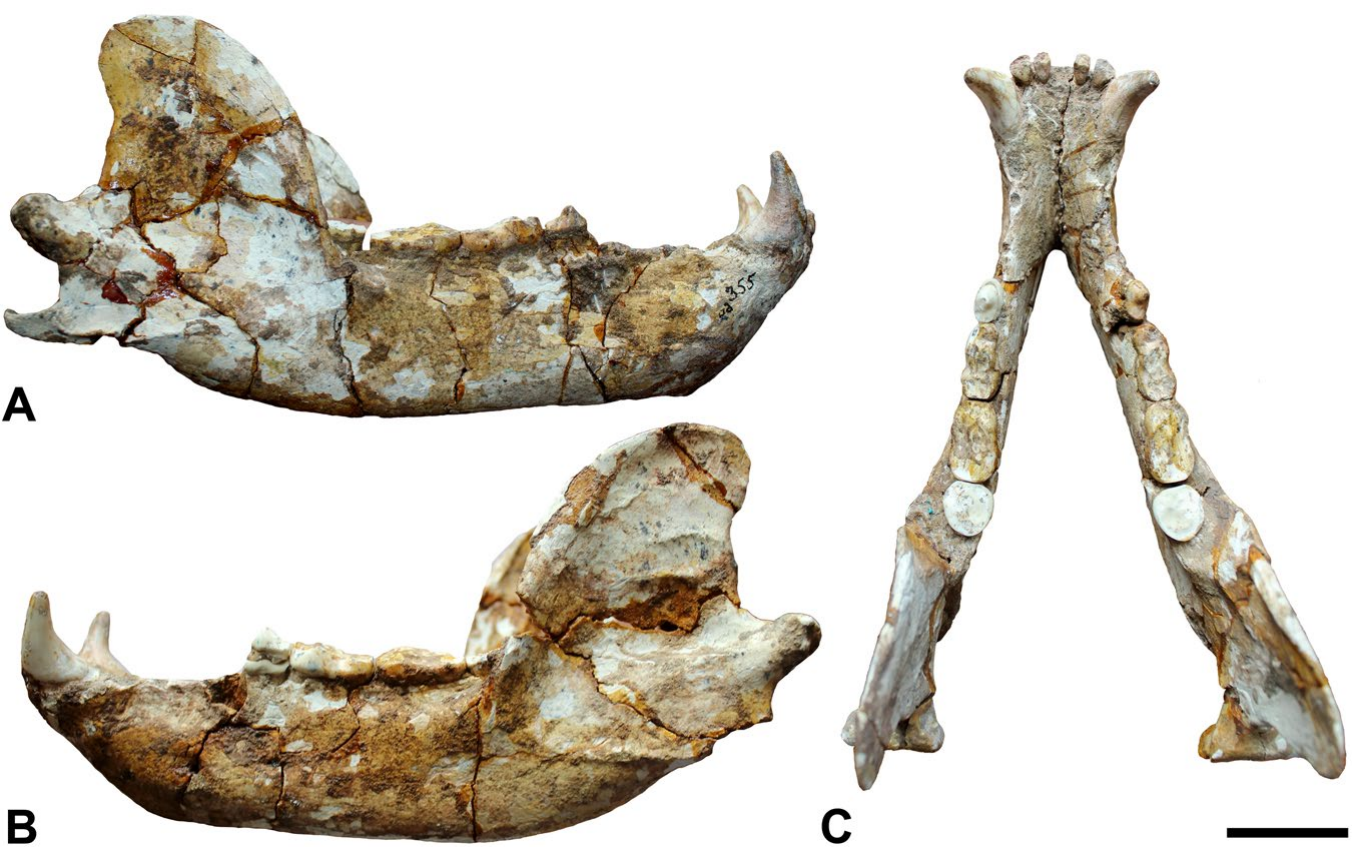
*Ursus etruscus* mandible (D355). (**A**) right buccal view, (**B**) left buccal view and (**C**) occlusal view. Scale bar = 5 cm.

The mandibular corpus displays a low profile in lateral view (e.g. D355, D1277, D1278: Figs. 2 and S2A). The mandibular condyle is laterally expanded, cylindrical and situated at about the alveolar level when the tooth row is oriented horizontally. D355 displays well-developed pterygoid insertions, which are distoventrally projected; the median pterygoid insertion extends distally and the mandibular ramus displays a vertical orientation in lateral view (Fig. 2A,B).

The incisors of the right (i1, i2 and i3) and left (i1) hemi-mandibular corpus D1278 are not well preserved (Fig. S2A–C). D36 shows incisors having single cusps that are not prominent. Cusp size increases from i1 to i3 (Fig. S2D–F).

The lower canines are highly dimorphic. Specimens D223, D2211, D355 and D36 have well-preserved small canines. In contrast, D1278, D1277 and D4940 are fully erupted lower canine teeth that are larger and show broken tip ends.

The lower anterior premolars (p1, p2 and p3) are brachydont, have a reduced size and are single rooted. The first premolar (p1) is slightly larger than the second (p2) and third (p3), showing a single prominent cusp that is elongated mesiodistally (Figs. 2C and S2C,F,I,L,O). It is placed close to c1 and there is a well-developed diastema between p2 and p3 (Figs. 2C and S2C,F,I,L,O).

The fourth premolars (p4) have two roots and a single vertically-oriented cusp, the protoconid (D223). Some of them show an eroded surface. D355 p4 shows a rounded cingulum. D4940 has small protruding cuspids on the mesial and distal sides of its cingulum (Fig. S3C–E).

The lower first molars (m1) have two roots and are mesiodistally elongated and buccolingually broader at the talonid region. Specimens D1277 and D1278 are slender than other specimens. The trigonid is differentiated from the talonid and is separated by a deep ridge (D2219, D4940, D3935), showing a constriction at the junction of the trigonid and talonid basin (Fig. S3A–E). The trigonid is dominated by a monocuspid protoconid, which is the main cusp. The paraconid is small, vertical and monocuspid, being situated at the mesial side. The metaconid is lower than the protoconid and is bicuspid, with the mesial cusp smaller than the distal one. The talonid is also bicuspid, with an unicuspid hypoconid and entoconid, but sometimes shows a hypoconulid (D5063, D1277, D1278; Fig. S3A–E).

The second molars (m2) have double roots and show a rectangular crown shape, with a trigonid well differentiated from the talonid basin by a clear ridge. The trigonid is composed of a monocuspid protoconid and a bicuspid or tricuspid metaconid (Fig. S3C–E). The talonid shows a well-differentiated hypoconid and a bicuspid entoconid. Both tooth sectors have a rounded and thick cingulum surrounding the molar crown.

The third molars (m3) have fused roots, showing a crown shape that ranges from rhomboid to oval. They are dominated by the trigonid, as the talonid basin is reduced. The protoconid is dominant and the paraconid is also differentiated in several specimens, although in most cases the lingual side is a cingulum formed by microcuspids. The talonid shows a small hypoconid and sometimes it is possible to differentiate the entoconid (e.g., D26 and D1020; Figs. 2CM and S2C,F,I).

In summary, the bear specimens from Dmanisi share a number of features with the Middle and Late Villafranchian populations of *U. etruscus* (e.g., the unicuspid metacone and deuterocone in the P4, the small, vertically oriented parastyle and a metastyle in the M1, the presence of lower and upper anterior premolars, the lack of paraconid in the p4, an unicuspid entoconid in the m1, and a bicuspid entoconid in the m2) and show also a number of derived features only present in later Late Villafranchian palaeopopulations like Orce (e.g., the presence of reduced anterior premolars with a mesiodistally enlarged diastema, an elliptic and buccolingually compressed shape of the p4, a metaconid with three cuspids and a trigonid blade that is buccolingually smaller than the talonid basin in the m2, and the rhomboid outline of the m3, which lacks indentations).

### Variability of tooth measurements

Table 1 shows the values of the coefficient of variation (CV, in percentage) obtained for the lower and upper teeth of seven extant ursid species. In all these species, the CV values of the upper and lower canine teeth tend to be higher (two or three-fold, on average) than those obtained for the cheek teeth. This indicates that sexual dimorphism among bears is particularly evidenced in the dimensions of the canines, as noted in earlier studies^37^. Specifically, the CV values of the canine teeth range between approximately 10 and 20% in all species. In the case of *U. arctos*, the most widespread species of Ursidae, these percentages tend to increase slightly for most teeth, which results from the fact that several populations (and subspecies) of this species were analyzed together. However, when the CV values are derived separately for each brown bear population from a single geographical locality, the estimates obtained tend to be similar to those of the other species.

**Table 1:** Coefficients of variation (in %) for measurements (L: mesiodistal length, B: buccolingual breadth) of the lower and upper teeth in skull samples of seven living bear species and in fossil populations of the extinct *Ursus etruscus* and *Ursus deningeri* from a number of Early and Middle Pleistocene sites of Europe.

SPECIES/SUBSPECIES (n)	cL	cB	p4L	p4B	m1L	m1B	m1TB	m2L	m2B	m3L	m3B	CL	CB	P4L	P4B	M1L	M1B	M2L	M2B
*Ursus maritimus* (6♀, 9♂, 2?)	12.6	12.3	5.5	7.3	3.2	5.0	4.2	3.0	4.3	9.3	7.4	13.9	14.6	7.0	8.5	6.3	4.1	7.4	5.3
*Helarctos malayanus* (2♀, 2♂, 8?)	9.9	11.5	7.7	10.5	4.4	8.0	8.1	6.3	8.8	9.9	23.4	19.2	12.8	9.4	6.4	4.7	6.5	7.7	6.0
*Melursus ursinus* (5♀, 4♂, 5?)	12.6	10.9	6.6	6.3	5.6	9.5	13.5	6.3	8.5	6.5	8.7	11.5	10.9	6.5	10.8	4.3	6.7	8.5	9.2
*Ailuropoda melanoleuca* (5♀, 2♂, 5?)	13.5	8.7	4.0	5.1	1.8	12.1	3.4	3.4	9.3	5.3	2.4	14.3	9.4	8.5	3.9	3.2	2.2	10.2	2.5
*Ursus arctos* (13♀, 14♂, 9?)	18.3	17.0	10.9	16.6	9.9	16.1	10.3	8.8	15.5	10.4	10.5	19.7	17.5	11.5	12.5	12.2	10.3	11.5	10.0
*U. arctos beringianus* (2♀, 1♂, 1?)	19.6	13.1	9.9	8.6	8.8	11.7	10.7	8.1	10.6	12.6	13.9	27.7	20.2	7.1	8.1	7.7	11.9	13.6	8.6
*U. arctos gyas* (1♀, 2♂)	10.8	11.2	8.2	4.6	4.9	29.4	6.2	3.0	2.5	7.7	8.4	12.3	24.4	13.7	13.8	31.5	16.1	29.0	15.7
*U. arctos horribilis* (2♀, 2♂, 3?)	14.6	17.2	9.1	10.7	8.1	10.6	10.7	9.4	11.1	10.0	8.6	16.0	14.4	13.5	11.2	7.5	9.5	6.6	8.2
*U. arctos isabelinus* (2♀, 2♂, 3?)	14.7	10.3	6.2	5.6	4.8	4.5	4.5	5.2	7.0	7.7	6.6	11.2	10.8	7.6	15.8	7.1	8.1	6.7	7.6
*U. arctos pruinosus* (2♀, 2♂)	15.5	11.3	10.4	25.3	7.2	8.3	7.7	4.9	6.8	8.7	6.6	8.1	9.7	7.0	7.1	4.7	3.2	2.7	4.4
*U. arctos syriacus* (2♀, 2♂)	13.2	9.4	6.1	2.3	4.2	2.8	3.7	7.0	2.8	2.3	3.8	28.8	26.1	16.7	18.1	8.6	14.7	15.1	13.8
*Ursus thibetanus* (4♀, 4♂, 6?)	12.5	14.8	12.7	8.9	9.6	32.3	8.6	6.4	20.6	12.8	8.8	12.3	14.4	9.3	12.3	7.8	8.5	9.7	8.0
*Ursus americanus* (4♀, 6♂, 3?)	12.7	11.2	7.5	7.0	6.2	8.0	6.4	7.3	17.6	8.9	8.0	14.4	13.9	7.9	8.6	7.1	5.6	7.4	7.6
*Ursus etruscus* All samples (135/121)	23.5	16.9	10.0	9.3	6.4	9.7	10.8	8.8	11.1	10.6	9.8	17.9	13.6	20.6	20.8	9.7	8.2	8.6	9.1
*U. etruscus* Saint Vallier (25/22)	9.4	8.3	7.4	1.9	3.6	4.5	5.0	3.3	2.4	1.8	3.3	12.6	12.5	8.9	14.8			7.9	9.1
*U. etruscus* Dmanisi (35/24)	14.2	13.5	9.1	7.8	6.3	11.7	13.7	9.1	11.2	8.1	6.7	12.9	17.4	28.2	23.3	5.3	8.1	12.5	5.1
*U. etruscus* Val d’Arno (44/29)	9.5	12.6	13.1	9.9	7.0		12.3	7.2		10.2	8.4	16.1	6.9	5.2	12.0	13.1	6.1	3.4	4.4
*U. etruscus* Olivola (11/4)	16.8	10.6	7.7	7.0	5.9		7.2	8.7		15.0	12.0	2.9	2.5	1.9	6.3	0.9	1.1	1.0	8.3
*U. etruscus* Pietrafitta (4/5)	7.6	8.9	4.1	3.0	0.4		5.3	4.0			0.0	9.6	9.4	3.6	10.0	3.2	4.1		
*U. etruscus* Pirro Nord (9/16)								6.6	4.5	12.4	9.2	2.7	4.2	4.0	10.3	6.9	7.1	5.3	
*U. etruscus* Orce (8/13)			1.3	2.0	2.7	5.9	3.3	2.0	6.0					5.7	3.0	2.7	4.6	6.7	4.2
*Ursus deningeri* All samples (217/146)	24.6	23.7	18.1	20.6	20.6	11.5	20.2	16.8	10.7	20.3	16.8	7.1	8.6	8.7	12.4	8.4	9.2	10.8	7.5
*U. deningeri* Untermassfeld (8)	3.2	5.2	5.0	3.8	5.1		8.5	3.2		4.3	10.5								
*U. deningeri* Trinchera Dolina (11/28)			9.2	7.6	4.7	6.9		5.1	4.7	11.7	6.9			6.9	5.6	3.8		5.6	
*U. deningeri* Vallonnet (128/92)	6.5	5.0	7.9	8.5	6.4	9.5	9.8	7.0	9.0	6.2	7.9			8.2	9.9	6.7	8.8	9.0	6.5
*U. savini* (4)	22.4	11.7	9.2	10.5	6.3		11.8	9.6		19.4	10.3								
*U. deningeri* Stránslá Skála (3)			9.3	9.8	5.1		7.1	3.6		11.8	9.2								
*U. deningeri* Aragó (47/22)	9.8	6.2	7.7	7.7	5.6	6.5	6.5	6.7	8.8	10.4	6.0	5.7	4.3	6.6	8.5	9.7	5.7	10.7	7.7
*U. deningeri* Château (4)	11.4	13.0	10.3	23.6	6.4		12.3	6.3		9.9	6.5								
*U. deningeri* Azé (4)			0.6	7.4	4.0		7.0	10.6		8.7	10.4								
*U. deningeri* Cueva Mayor (4)	10.0	4.6	18.5	25.7	7.2		15.3	11.6		15.7	12.6								
*U. deningeri* Sima de los Huesos (4/4)	18.7	17.7	15.5	7.8	5.0		7.1	8.0		12.8	5.8			8.8	16.5	4.5	12.0	9.0	9.4

Abbreviations: c, lower canine; p, lower premolar; m, lower molar; C, upper canine; P, upper premolar; M, upper molar. Sample sizes in the case of extant species indicate the number of sexed individuals; for fossil sites, they refer to the lower and upper dentition. Data from^33,38,40,42,77–81,84^ and J.M.-M., B. F. (unpublished data).

Concerning the extinct species, the CV values of the pooled sample of *U. deningeri* are similar to those of all modern bears. In contrast, the values derived from the joint analysis of all populations of *U. etruscus* increase in the case of the lower canine teeth and also, but to a lesser extent, in the upper canines and the upper and lower cheek teeth. However, the CV values obtained when the fossil populations of *U. etruscus* are analyzed separately are similar to those of extant bear populations, even in the case of the Val d’Arno sample, which comprises several sites (and is presumably time-averaged). The CV estimates obtained for Dmanisi are similar for most teeth to those from other populations of both modern and fossil bears, including Val d’Arno. Given that such amount of variability is not surpassed by the fossil sample of Dmanisi, this suggests that only one ursid species was present at this Georgian site and that this palaeopopulation was not time-averaged. In the case of the canine teeth, however, the CV estimates for Dmanisi are higher than those for other populations of *U. etruscus* except Olivola. Given that sexual dimorphism in bears is particularly evident in the dimensions of these teeth^37^, this suggests a high level of dimorphism in the Dmanisi palaeodeme (as happens also in the case of the hominin population). This probably explains why Vekua suggested that two species (*U. etruscus* and *Ursus* sp.) were present at Dmanisi^15^, and that later both Baryshnikov^16^ and Wagner^17^ included these remains in a separate subspecies (*U. etruscus vekuai*).

### Principal components analyses and canonical variates analyses

**Figure 3:**
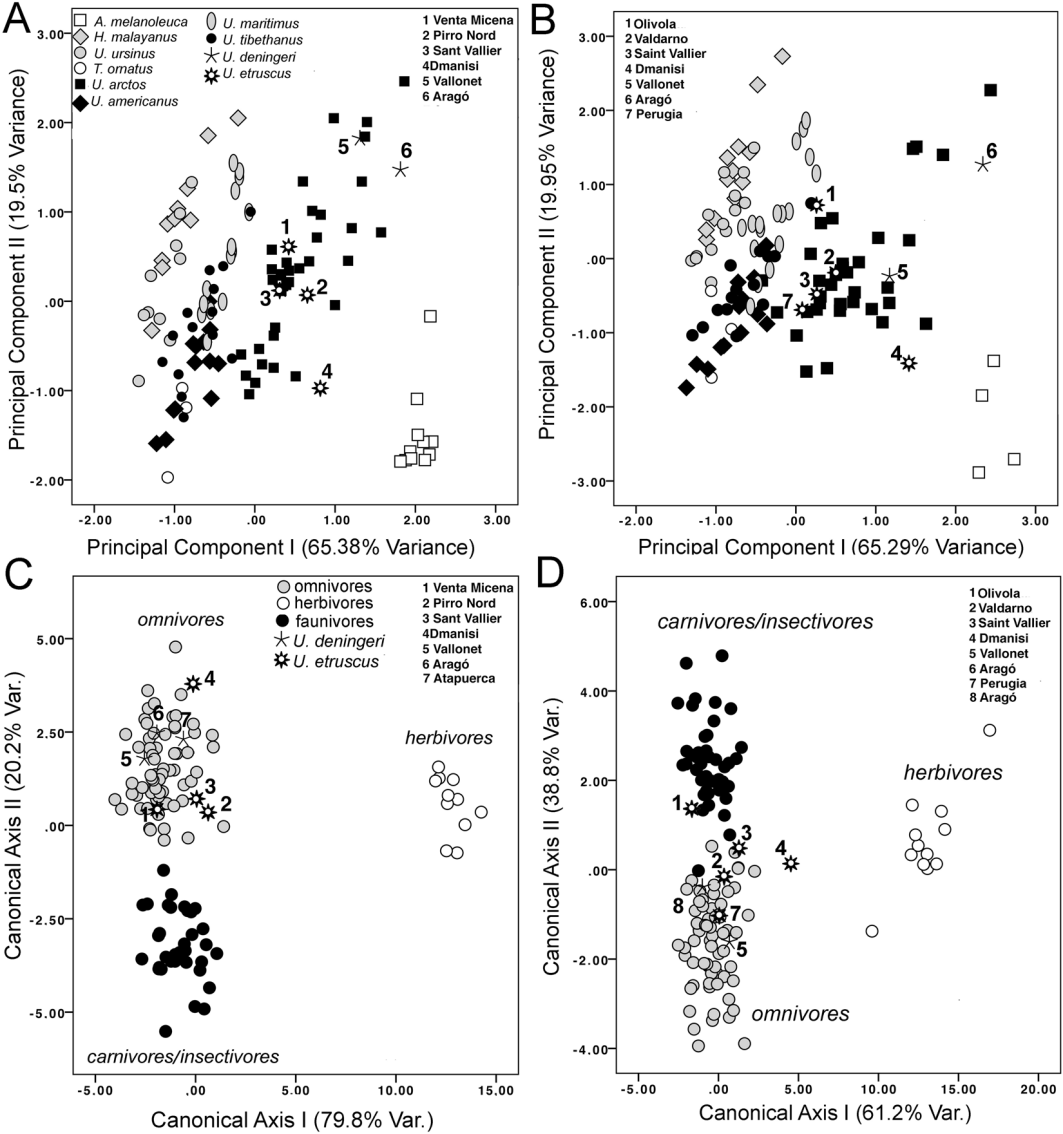
Morphometric analyses of tooth measurements in extant and extinct bears. (**A**) Bivariate scatterplot of the specimens’ scores on the two first principal components for the lower (mandibular) teeth. (**B**) Bivariate scatterplot of the specimens’ scores on the two first principal components for the upper (maxillary) teeth. (**C**) Bivariate scatterplot of the specimens’ scores on the two first canonical functions for discriminating among dietary types in ursids with the tooth measurements of the lower dentition. (**D**) Bivariate scatterplot of the specimens’ scores on the two first canonical functions derived from the upper dentition. The points for *U. etruscus* and *U. deningeri* correspond to the sample means of the sites studied. Numbers indicate for the sites of the fossil specimens.

Only the first two PCs provided by the PCA performed with the variables that measure the dimensions of the lower teeth showed eigenvalues greater than one (i.e., they subsumed more variance than the one explained by any single tooth variable). The scores of the extant and extinct bears on the morphospace defined by these PCs, which jointly account for 85% of the original variance, are shown in Fig. 3A. Given the factor loadings of the tooth measurements in these PCs (Table 2), PC1 can be considered as a “size-vector” with all cheek teeth showing high positive loads on it. In contrast, the canine dimensions are the only variables that take high loadings on PC2 while most others score negatively. For this reason, PC2 measures the size of the lower canines relative to other teeth. According to the dietary preferences of the living bears^37,38^, PC1 describes a dietary gradient among ursids, because it separates the giant panda (*Ailuropoda melanoleuca*), the only truly herbivorous bear, which takes high positive values on this component, from the Malayan sun bear (*Helarctos malayanus*) and the sloth bear (*Melursus ursinus*), both with a diet heavily based on insects, followed by the polar bear (*Ursus maritimus*), a species that only consumes flesh^37,38^, which all take negative projections. Strikingly, PC2 seems to reflect in part sexual dimorphism, as the variables with higher positive loadings on this eigenvector are the length and width of canines (cL andcB, respectively; Table 2). This means that the specimens with positive scores on this second axis (mostly male individuals) tend to have comparatively larger canines, while those scoring negatively (mostly female individuals) are characterized by the opposite condition (Table 2). However, this gradient applies also to the size of the canines relative to the cheek teeth in the species analyzed: the ursids that rely more on animal resources have proportionally larger canines, as reflected in their higher values on PC2, than those with a more herbivorous diet, which score negatively. The specimens of both *U. etruscus* and *U. deningeri* take intermediate scores on PC1 and PC2, plotting with the living omnivorous bears. However, *U. deningeri* tends to score more positively on PC2 than *U. etruscus*, which reflects that the former had relatively larger canines than the later.

**Table 2:** Factor loadings and communalities (Communal.) of all teeth measurements obtained in the first two principal components (PCI, PCII) derived from the upper and lower dentitions.

Variables/PCs	Lower teeth			Upper teeth		
	PCI	PCII	Communal.	PCI	PCII	Communal.
CL	0.136	0.956	0.932	0.259	0.883	0.848
CB	0.265	0.928	0.932	0.291	0.871	0.843
P4L	0.874	−0.196	0.802	0.931	−0.042	0.868
P4B	0.909	−0.114	0.840	0.925	−0.138	0.874
M1L	0.975	−0.032	0.953	0.912	−0.16	0.831
M1B	0.875	−0.199	0.805	0.929	−0.090	0.872
M2L	0.885	0.148	0.806	0.893	−0.092	0.806
M2B	0.884	−0.191	0.818	0.953	−0.142	0.929
M3L	0.825	0.156	0.705			
M3B	0.947	0.014	0.898			
Eigenvalues	6.538	1.951		5.274	65.923	
% variance	65.384	19.506		1.596	19.955	

The PCA performed with the variables that measure the upper teeth provided also two PCs, the first ones, with eigenvalues greater than one (Table 2). These eigenvectors accounted jointly for more than 85% of the original variance. Figure 3B shows the scores for the extant and extinct bears on the morphospace defined by them. Again, the factor loadings of the tooth measurements show that the dimensions of the cheek teeth contribute more to PC1, which can be interpreted as a size vector, while PC2 is clearly a shape vector in which the size of the canines load positively while all other tooth dimensions score negatively (and weakly). This indicates that PC2 estimates in part the degree of sexual dimorphism in ursids, as happened with the lower dentition, and also reflects the relative importance of animal resources on the bear diet. As in the preceding case, the morphospace depicted by these two PCs describes a dietary gradient from the herbivorous bears, which all take positive loadings on PC1 and negative ones on PC2, to those with an insectivorous or carnivorous diet, which take negative loadings on PC1 and positive ones on PC2 (Fig. 3B). Again, the specimens of *U. etruscus* and *U. deningeri* plot on an intermediate region of this morphospace that is occupied by the living bears that have an omnivorous diet.

The canonical variates analysis performed to find those features of the lower teeth that best distinguish among the three dietary groups of ursids (i.e., herbivores, omnivores and faunivores) yielded two functions (Table 3), which correctly classified more than 98% of the living specimens using the leave-one-out cross-validation procedure. While the first function (λ = 18.438; Wilks’ Lambda = 0.009; d.f. = 16; P < 0.0001) distinguishes the herbivorous bears, the second one (λ = 20.2; Wilks’ Lambda = 0.176; d.f. = 7; P < 0.0001) separates the omnivores from the faunivores among the rest of the sample (Fig. 3C), according with a set of morphological traits (see Table 3). As expected, all fossil specimens of *U. etruscus* as well as of *U. deningeri* are classified as omnivores, as they score within the range of variation of living omnivorous bears.

**Table 3:** Standardized coefficients of those teeth measurement included in the canonical functions (CFI, CFII) obtained from the analyses of lower and upper teeth separately and using a step-wise procedure.

Lower teeth			Upper teeth		
Variables	CFI	CFII	Variables	CFI	CFII
CB	−0.271	−0.511	CL	−0.109	0.024
P4L	0.431	−0.494	CB	−0.164	0.342
P4B	0.586	−0.351	P4L	0.361	0.355
P4B	0.412	0.049	M1B	0.499	0.387
M1L	0.412	0.049	M2L	−0.408	−0.288
M1B	0.164	0.147	M2B	0.532	−0.483
M2L	−0.968	0.435			
M2B	0.226	0.027			
M3B	0.223	0.498			
Constant	−1.759	−3.110		−4.631	−0.323
Eigenvalues	18.438	4.672		6.105	3.866
% variance	79.8	20.2		61.2	38.8

Similarly, the canonical variates analysis performed on the upper teeth of living and extinct bears to distinguish among the same dietary groupings (Table 3) yields two canonical functions that correctly classify ~95% of the living specimens using the leave-one-out cross-validation procedure. The first function distinguishes (λ = 6.105; Wilks’ Lambda = 0.029; d.f. = 12; P < 0.0001) herbivorous bears from the rest, while the second (λ = 3.866; Wilks’ Lambda = 0.206; d.f. = 5; P < 0.0001) separates the omnivores from the faunivores (Fig. 3D). As in the case of the lower dentition, all fossil bears are within the range of shape of variation of the living omnivores with the only exception of the sample of *U. etruscus* from Olivola (Italy), which occupies an intermediate portion of the morphospace between the omnivores and faunivores.

### Tooth microwear analysis

The bivariate plot (Fig. S4) depicted from the scores on the first two eigenvectors (>90% of the original variance) obtained from the PCA of tooth microwear data indicates a very low intraspecific variability for the bears of Dmanisi compared to other ursid species included in the analysis. This result was in part unexpected because the bears from Dmanisi exhibit a high variability in tooth shape, which could presumably translate in palaeodietary differences among the specimens. Moreover, the low dietary variability estimated for these bears suggests that the palaeopopulation of Dmanisi was not time-averaged; otherwise, the expectations would be to find substantial variability in tooth microwear patterns resulting from changes in climate and resource availability through time.

The comparison of the Dmanisi scores with those for other specimens of *U. etruscus* from Orce and the extant *U. arctos* and *U. maritimus*, shows that the density of pits and gouges better discriminates their dietary habits. Most specimens of these species record large pits (LP), cross scratches (XS) and puncture pits (Npp). Evidence of hyper-coarse scratches is scarce and gouges are totally absent, with the only exception of the hypercarnivorous *U. maritimus*.

The bears from Dmanisi score an average density of 199.1 pits/mm^2^ and 170.0 scratches/mm^2^ (Table 4). These values are slightly higher than those recorded in the specimens from Venta Micena, Barranco León and Fuente Nueva-3 (182.2 pits/mm^2^ and 146 scratches/mm^2^), which plot close to the fossils from Dmanisi in the PCA-plot. Compared to the extant species (Table 5), the average density of scratches is higher in Dmanisi than in both the hypercarnivore *U. maritimus* (69.89 scratches/mm^2^) and the omnivore *U. arctos* (117.84 scratches/mm^2^). This could result from a diet that included in *U. etruscus* more silica-rich plant resources. To a lower extent, this difference applies also to the density of pits in *U. arctos* (155.44 pits/mm^2^), which is lower than in the bears from Dmanisi, although this species plots near the extinct *U. etruscus* from Dmanisi and Orce. However, the density of pits in the hypercarnivore *U. maritimus* (328 pits/mm^2^) is higher than in the fossil sites, which explains why this species plots distant from all ursids in the PCA-plot.

**Table 4:** Microwear results of *Ursus etruscus*, with average mean density of pits (DP), average mean density of scratches (DS), cross scratches (XS) and Gouges (G), Scratch Width Score (SWS), large pits (LP), number punctuate pits (Npp) and number of hypercoarse scratches (Nhyper_cs) from Dmanisi.

Spm. No	Tooth	DP (Mean)	DS (Mean)	XS	G	SWS	LP	Npp	Nhyper_cs
D355	p4	209.375	156.25	1	0	1	1	1	1
D 218	p4	203.125	146.875	1	0	1	1	0	1
D36	p4	203.125	181.25	1	0	2	0	0	1
D36	p4	193.75	178.125	1	0	0	0	0	0
D2215	P4	203.125	165.625	1	0	0	1	0	0
D1277	m1	203.125	159.375	1	0	2	1	1	0
D1278	m1	181.25	153.125	1	0	2	1	1	0
D5063	m1	190.625	150	1	0	1	1	1	0
D2219	m1	209.375	190.625	1	0	0	1	1	0
D4940	m1	193.75	190.625	1	0	1	1	1	0
D2211	m1	196.875	171.875	1	0	0	1	1	0
D2211	m1	203.125	143.75	1	0	0	1	1	0
D3935	m1	215.625	153.125	1	0	0	1	1	0
D 218	m1	206.25	162.5	1	0	1	1	0	1
D50	M1	190.625	178.125	1	0	1	1	1	0
D2214	M1	178.125	171.875	1	0	1	1	0	1
D1277	m2	193.75	153.125	1	0	0	1	1	0
D5063	m2	209.375	190.625	1	0	1	1	1	0
D1029	m2	190.625	168.75	1	0	1	1	1	1
D4940	m2	200	156.25	1	0	0	1	0	0
D2219	m2	212.5	193.75	1	0	0	1	0	0
D1394	m2	203.125	200	1	0	0	1	1	0
D2211	m2	203.125	168.75	1	0	1	1	1	0
D2211	m2	200	181.25	1	0	1	1	1	0
D 218	m2	209.375	187.5	1	0	1	1	1	1
D2533	M2	221.875	175	1	0	2	1	2	1
D52	M2	193.75	159.375	1	0	2	1	1	1
D4713	M2	190.625	175	1	0	1	1	1	0
D1029	m3	181.25	184.375	1	0	1	1	0	1
D5063	m3	206.25	171.875	1	0	1	0	0	0
D5355	m3	193.75	153.125	1	0	1	0	0	1
D1277	m3	200	162.5	1	0	1	0	0	1
D355	m3	187.5	168.75	1	0	1	1	1	1
D2219	m3	187.5	165.625	1	0	1	1	1	1
D2211	m3	203.125	168.75	1	0	0	1	1	0
Average (N = 35)	199.11	170

**Table 5:** Microwear results of extinct and extant ursid species.

Species	Material	Number	DP	DS	XS	G
*U. etruscus*	VM_10316		190.625	118.75	1	0
*U. etruscus*	VM_10309		175	128.125	1	0
*U. etruscus*	VM_10311		184.375	131.25	1	0
*U. etruscus*	VM_10315		159.375	128.125	1	0
*U. etruscus*	VM_10313		165.625	165.625	1	0
*U. etruscus*	VM_10314		153.125	131.25	1	0
*U. etruscus*	VM_10318		203.125	131.25	1	0
*U. etruscus*	VM_10319		190.625	134.375	1	0
*U. etruscus*	VM_3117		212.5	159.375	1	0
*U. etruscus*	FN3 02 U96 N9 4		187.5	178.125	1	0
*U. etruscus*	FN3 01 Q92 UME1 11		140.625	146.875	1	0
*U. etruscus*	FN3 95 T8d AB h		193.75	175	1	0
*U. etruscus*	BL03 J52 UME 14 N118		212.5	178.125	1	0
Average		13	182.2	146.63		
*U. arctos*	MCZ 39655		393.75	93.75	1	0
*U. arctos*	MCZ 35948		231.25	137.5	1	0
*U. arctos*	MCZ 7373		325	81.25	1	0
*U. arctos*	MCZ 7374		156.25	37.5	1	0
*U. arctos*	MCZ 30644		268.75	137.5	1	0
*U. arctos*	Gemeinde Alland		56.3	168.8	1	0
*U. arctos*	NWA		181.3	78.1	1	0
*U. arctos*	NWA		168.8	56.3	1	0
*U. arctos*	SMNS		46.9	115.6	1	0
*U. arctos*	NHMW		65.6	115.6	1	0
*U. arctos*	NHMW		92.2	146.9	1	0
*U. arctos*	NHMW		90.6	156.3	1	0
*U. arctos*	NHMW		43.8	190.6	1	0
*U. arctos*	NHMW		131.3	140.6	1	0
*U. arctos*	SZ		175.0	81.3	1	0
*U. arctos*	SZ		168.8	75	1	0
*U. arctos*	NHMW		46.9	190.6	1	0
*Average*		17	155.44	117.84		
*U. maritimus*	MCZ 5004		393.75	43.75	1	1
*U. maritimus*	MCZ 7416		400	62.5	1	1
*U. maritimus*	MCZ 9328		387.5	118.75	1	1
*U. maritimus*	MCZ 9329		300	75	1	1
*U. maritimus*	MCZ 11769		250	87.5	1	1
*U. maritimus*	MCZ 29684		275	75	1	1
*U. maritimus*	MCZ *30237		337.5	81.25	1	1
*U. maritimus*	MCZ *30237		318.75	43.75	1	1
*U. maritimus*	MCZ 30238		381.25	43.75	1	1
*U. maritimus*	MCZ 46532		237.5	81.25	1	1
*U. maritimus*	MCZ 46533		331.25	56.25	1	1
*Average*		11	328.41	69.89		

With average density of pits (DP), average density of scratches (DS), cross scratches (XS) and gouges (G). *Ursus etruscus* from Orce sites (Venta Micena (VM). Barranco León (BL) and Fuente Nueva-(FN3) and extant large mammal taxa: *Ursus arctos* from Münzel *et al*.^26^, *Ursus maritimus* from Dewar^64^.

**Figure 4:**
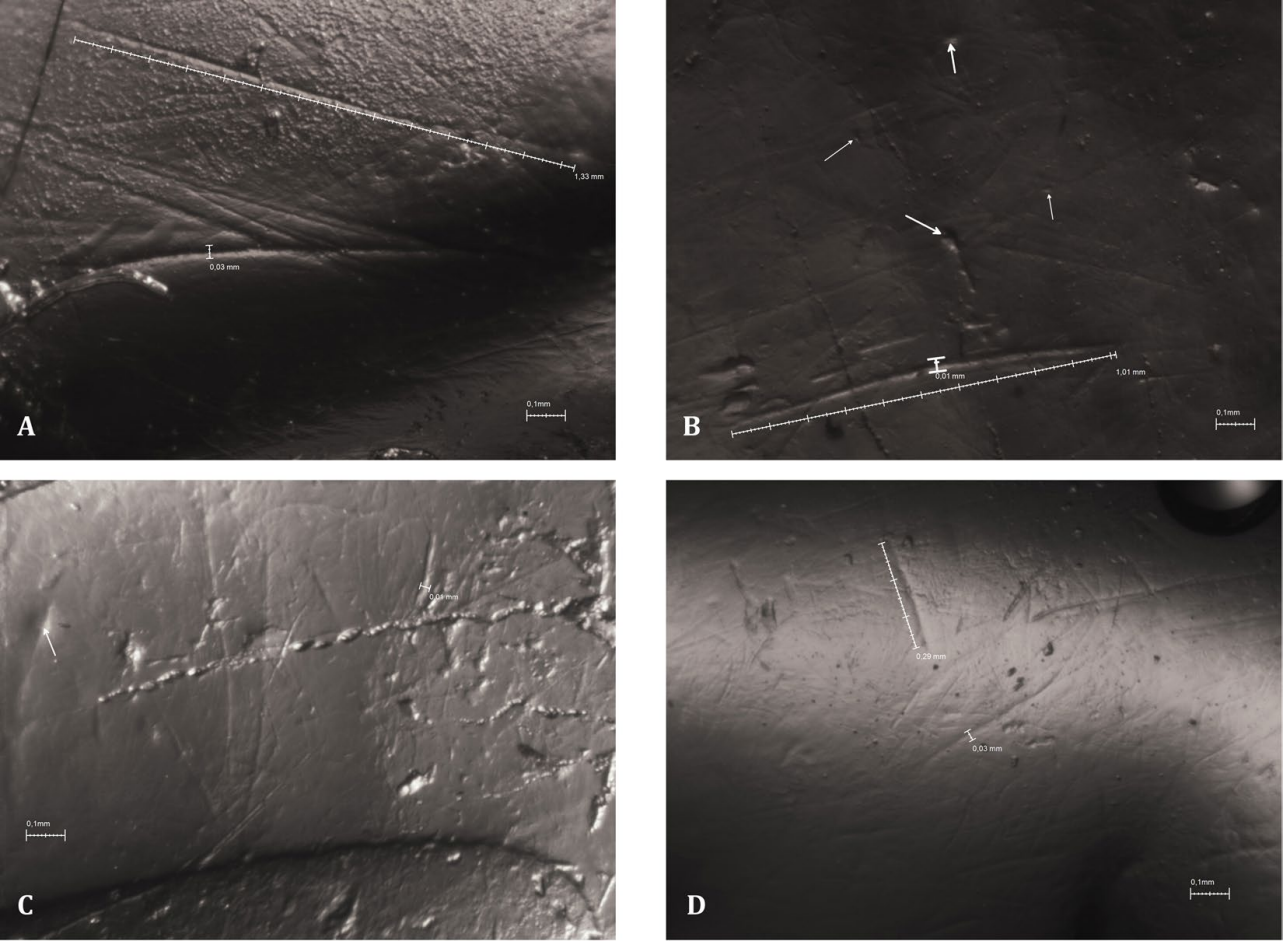
A microwear features of grinding and slicing facets of *Ursus etruscus* from Dmanisi. (**A**) Slicing facet of a left lower p4 (D36). (**B**) slicing facet of a left m3 (D355). (**C**) grinding facet of a left m1 (D1277) and grinding facet of left m2 (D4940).

There are differences in the density of pits and scratches on the grinding and slicing facets of the carnassial teeth from Dmanisi (Fig. 4), as happens in the other bears. The slicing facet scores a higher density of scratches than of pits, showing longer scratches and also cross-scratches, which are shorter and fainter. Conversely, the grinding facet shows a higher density of pits than of scratches. Most of these pits are small, although there are few medium-to-large sized pits. Pit diameter ranges from 0.02 to 0.08 mm. The scratches on the grinding facet are shorter than on the slicing facet, ranging in length from 0.12 to 0.67 mm. In contrast, the slicing facet of the fossil *Ursus* species preserves scratches with a length range from 0.12 to 1.33 mm. Pit diameter ranges from 0.02 to 0.08 mm. Importantly, most scratches and cross scratches are observed on the slicing facet in all taxa, which indicates the functional emphasis on this tooth region for processing plant stuffs.

## Discussion

### Systematics and biochronology

Vekua^15^ was pioneer in describing the large mammals assemblage of Dmanisi. In his original study, he assigned the ursid specimens to two different species: *U. etruscus* and *Ursus* sp. However, later, Baryshnikov^16^ and Wagner^17^ included all Dmanisi remains in the subspecies *U. etruscus vekuai*.

*Ursus etruscus* is the typical ursid species recorded in the faunal assemblages of Eurasia during the Middle and Late Villafranchian^16,17,31,39–41^, and was described originally on the basis of the specimens from Figline (Upper Valdarno, Italy). According to the revisions made by Mazza and Rustioni^35,42^, Baryshnikov^16^ and Wagner^17^, the specimens of the former species are found during the Late Villafranchian, being last recorded in the Orce localities (ca. 1.6-1.3 Ma^12,31,36^). However, the specimens from the Middle Villafranchian sites (e.g., Saint Vallier [France], Kuruksay [Tajikistan] or Puebla de Valverde [Spain]) show some differences and were ascribed to *Ursus* aff. *etruscus* by Mazza and Rustioni^42^, or to a different subspecies of *U. etruscus* by Baryshnikov^16^. In fact, Baryshnikov^16^ in his extensive revision of the family Ursidae included all known *U. etruscus* specimens in four subspecies^16^ (G. Baryishnikov, pers. comm. to J.M.-M. 13/7/2017): (i) *U. e. verescagini* Sharapov, 1986 including the localities of Kuruksai, Obi-Garm and Tutak (Tajikistan, MN17, Middle Villafranchian), which is characterized by the presence of large teeth, M1 and M2 comparatively wide, m1 with a monocuspid or bicuspid metaconid, and m2 with a moderately long talonid; (ii2) *U. e. saintvallierensis* Baryshnikov, 2007 including the localities of Saint-Vallier and La Puebla de Valverde (MN17, Middle Villafranchian), characterized by teeth smaller than those of *U. e. verescagni*, M2 with a narrow talon, m1 with a bicuspid metaconid, and m2 with a short talonid; (iii) *U. e. vekuai* Baryshnikov, 2007 including all the known specimens from Dmanisi (Late Villafranchian), with teeth larger than in the other subspecies, of which the M2 is particularly enlarged, and m1 with a bicuspid metaconid; and (iv) *U. e. etruscus* Cuvier, 1823 including the localities of Olivola, Figline, Farneta and Pietrafita (Late Villafranchian), characterized by smaller anterior premolars and posterior molars larger than in *U. e. saintvallierensis*, M2 with a comparatively large talon, m1 with a bicuspid metaconid, and m2 with a long talonid.

In general terms, we agree with Baryshnikov^16^ and Wagner^17^, who included all known bears from Dmanisi in a single species, *U. etruscus*, and justified the known variability on the basis of sexual dimorphism, as tested in this study. However, according to our opinion, the morphometric and morphological variability displayed by the specimens of *U. etruscus* from the Middle and Late Villafranchian sites of Europe is not enough for separating them among several subspecies (contra. Mazza and Rustioni^35,42^, Baryshnikov^16^). Furthermore, we do not agree with Mazza and Rustioni^35,42^ and Wagner^17^ in considering *U. etruscus* as a highly specialized species without descendants. In contrast, we think that the lineage *U. etruscus-U.deningeri-U.spelaeus* is well documented during the Pleistocene, as we already stated in previous works^31,40^.

As noted above, we consider the ursid remains from Dmanisi as *Ursus etruscus* on the basis of the following characters shared with the Middle and Late Villafranchian ursids previously described as *U. etruscus*^16,17,31,35,39–43^: (i) P4 with an unicuspid metacone and deuterocone; (ii) M1 with a slightly developed and vertically oriented parastyle and a metastyle; (iii) presence of lower and upper anterior premolars; (iv) p4 without paraconid; (v) m1 with an unicuspid entoconid; and (vi) m2 with a bicuspid entoconid.

Finally, the Dmanisi specimens display several derived features not present among the Middle Villafranchian forms^43^ and more frequently recorded in the later Early Pleistocene forms (e.g., the Orce localities^31^; J.M.-M. unpublished data), namely: (i) anterior premolars present but reduced, specially p3; (ii) a mesiodistally enlarged diastema between the anterior premolars; (iii) an elliptic and buccolingually compressed lower p4 without cuspulids; (iv) m2 with a metaconid showing three cuspids and a trigonid that is buccolingually smaller than the talonid; and (v) m3 with a rhomboid outline without indentations. As we already stated before, the former morphological characters, present in the Late Villafranchian forms but not seen in the Middle Villafranchian ones, reinforce the idea of gradual evolution in the cave bear lineage (*U. etruscus-U. deningeri-U.spelaeus*) (contra. Mazza and Rustioni^42^).

### Palaeoecology

Using a multiproxy of palaeobotanical analyses (phytoliths, pollen grains and carpo-remains), Messager^44^ recently addressed the paleoecological scenario of Dmanisi at 1.8 Ma. This study suggests the presence of a predominantly temperate vegetation dominated by C3 grasses, which reflect an increase in aridity at the middle part of the stratigraphic sequence^44,45^. Moreover, the presence of an open and dry environment in Dmanisi, with large patches of forested areas, is also supported by faunal data^4,15,46–48^ (large and small vertebrates). However, it must be noted that phytolith analysis is biased as it records only the presence of herbs, which probably overestimates the extent of open habitats in Dmanisi. This contradicts the environmental inference derived from the composition of the ungulate assemblage, largely dominated by deer (closed habitat dwellers). Also, it is worth noting that E. Kvavadze found in a coprolite a variety of seeds and a wide pollen spectrum of trees, shrubs, grasses and herbs^49^, which indicates the presence of a patched habitat. These grassland and forest steppe dominated ecosystems probably played a significant role in maintaining a rich and diverse palaeocommunity of large mammals^45^. Similarly, an open habitat with patches of forested areas has been inferred for the Early Pleistocene site of Venta Micena^50,51^, which is slightly younger in age than Dmanisi. A recent palaeosynecological study of the assemblage of large mammals preserved at Venta Micena was based on a mathematical model that allows to quantify: (i) the biomass of primary consumers available to the members of the carnivore guild; and (ii) the pattern of resource partitioning and competition intensity among the secondary consumers. The results obtained^10^ showed that although the biomass of ungulates available to the carnivores was lower than the value expected under optimal conditions, more than half the individuals and biomass of secondary consumers expected were reached, which indicated the presence of a viable ecosystem in Venta Micena. A study in progress with this model of the large mammals assemblage of Dmanisi suggests that the ecological conditions in this site were similar to those in Venta Micena, although it should be noted that the carnivore guild at Dmanisi includes a species, the giant cheetah *Acinonyx pardinensis*, which is not recorded at Venta Micena, a site in which more than 25,000 fossils of large mammals have been unearthed after decades of systematic excavations (which strongly argues against taphonomic bias). This suggests increased levels of competition intensity among the members of the carnivore guild of Dmanisi, which could explain the hypocarnivore condition deduced for *U. etruscus* in this study. Given that intraguild competition and predation plays a prominent role in shaping ecological communities^52,53^, the results for *U. etruscus* agree with the flexible dietary behaviour depicted by modern *U. arctos*, a generalist omnivore, as a function of resource availability and competition intensity with other predators, including humans^54–57^.

The analyses of tooth microwear and dental morphometrics performed here on the specimens of *U. etruscus* suggest that the bear species that coexisted with the first hominin populations that inhabited Eurasia was clearly opportunistic and had an omnivorous behavior, feeding on both plant resources and vertebrate flesh depending upon availability. Isotopic data from Venta Micena indicates that *U. etruscus* recorded high levels of δ^15^N, which suggests that a regular consumption of fish from the moderately saline lacustrine systems present at the Orce basin of the Baza and Guadix depression^58^ could have played a significant role in the diet of *U. etruscus*^51^. Moreover, although these high δ^15^N values could also have resulted in part from a prolonged hibernation^59^, climatic reconstructions for the Orce sites suggest a milder and less seasonal climate than in present times in the surroundings of the lacustrine areas, which were fed by thermal springs, thus arguing against hibernation^60,61^. Unfortunately, there is no isotopic study of the Dmanisi fauna available, which precludes substantiating a biogeochemical comparison with Venta Micena.

Brown bears have a diet that normally includes <20% of flesh^37,54,62,63^, hunting only opportunistically with a seasonally-driven diet^64^. Accordingly, as the microwear data presented here for the population of *U. etruscus* from Dmanisi is similar to the extant *U. arctos*, we infer that this species might have consumed similar dietary resources to those exploited by the living brown bear.

On the other hand, the average density of scratches and pit diameter of *U. etruscus* from Dmanisi are similar to the values estimated in the specimens from the Orce sites^31^, but some teeth from Dmanisi score longer scratches on the slicing facet, which could evidence a greater consumption of grass. The high density of pits in *U. etruscus* could be related to a larger intake of dust and grit during rooting on areas with a low vegetation cover. In fact, although the presence of hypsodont (i.e., high crowned) teeth in ungulates has been traditionally interpreted as an adaptation for grazing on herbaceous plants with abundant silica-rich phytoliths, a comparative study^65^ showed that it correlates better with a foraging behavior in open habitats, where hypsodont teeth represent an adaptation against tooth wear resulting from the airborne grit and dust accumulated on the herbaceous plants that grow at low levels above the ground. Bear teeth are not hypsodont like those of grazing ungulates, but the effects of a diet with an important contribution of succulent grasses would be similar on the grinding surface of the teeth of *U. etruscus*.

The omnivorous dietary behaviour deduced for *U. etruscus* in this study provides a key for explaining the coexistence of this species with early *Homo* out of Africa.

Table S1 shows the record of Villafranchian *U. etruscus* in Europe, Central Asia and the Levantine Corridor, together with the earliest presence of *Homo* in this region. Two largely omnivorous species of the large mammals fauna, one carnivore (*U. etruscus*) and one primate (*Homo* sp.), coincided in time and space since the first human dispersal out of Africa, as dated at 1.8 Ma in Dmanisi. Coincidentally, another large omnivore species, *Sus strozzii*, which is well known in the European fossil record, disappeared at the same time in Europe, although different suid species persisted in Asia, the Levantine Corridor and Africa. Europe was empty of pigs during more than 600 Ka, until the arrival of *Sus scrofa* around 1.2 Ma, which marks the beginning of the Epivillafranchian^66^ and is first recorded at the level TE9 of Sima del Elefante in Atapuerca, Spain^67^. The extinction of a large omnivorous species, a suid, in Europe in coincidence with the arrival of other large omnivorous species, a primitive form of *Homo erectus* (i.e., a primate), can be tentatively related with a direct competence among omnivorous species in the middle latitudes.

Omnivory, the consumption of both animal and plant tissues, represents the adaptive benefits of being a non-specialist and is an excellent dietary strategy for obtaining a correct mix of nutrients. However, it is also a difficult lifestyle in terms of morphological and behavioral adaptations^68^. For this reason, omnivory is relatively rare among large mammals, with the exception of primates and, to a lesser extent, suids among artiodactyls and ursids among carnivores. Although acknowledging that this dietary category is so general that it collapses substantial variability in the actual dietary niche occupied by each species, a huge evidence indicates that the earliest humans were largely omnivorous, exploiting a diverse array of plant stuffs and an important component of animal resources based on a scavenging behavior that developed in Eastern Africa^6–9,69–72^. After their arrival into the middle latitudes, hominins faced a more seasonal climate than in the environments of subtropical Africa, which resulted in that few vegetable resources were accessible during several months of the year, especially in winter. For this reason, human survival at the temperate climate of the Caucasus probably relied more heavily on a scavenging strategy than at lower latitudes in East Africa, which amplified the direct competence with large carnivores, especially the giant, short-faced hyena *Pachycrocuta brevirostris*^8–10,13,73^, and also with other omnivorous species, like suids and ursids. In the latter case, competition was not only for carrion but also for other very important resources in winter, for example nuts^74^. Moreover, it has been argued that adaptations for food gathering and dental processing of belowground plants testify to the early convergence of bears, pigs, and humans on this resource, which can represent a substantial dietary contribution for these omnivorous animals in temperate and tropical grasslands, especially in highly seasonal climates where the belowground biomass is relatively stable^75^.

In this ecological scenario, the extinction of pigs in Europe coincided with the first arrival of the genus *Homo* and also with the survival of *U. etruscus*. Therefore, suid extinction can be tentatively linked to increased competence among omnivores for food resources, especially vegetables. These three taxa have bunodont check teeth well suited for an omnivorous diet, but the scavenging behavior in pigs is really anecdotal if we compare it with the large hyenas and humans, which both have bone-cracking abilities using their heavily built premolar teeth or the stone tools, respectively. Suid specialization in nut eating (e.g., acorns, nuts, chestnuts, hazelnuts, almonds or pistachios), which is typical for the Mediterranean environments, also falls in direct competence with humans, especially during autumn and winter. There are other feasible scenarios for the disappearance of pigs from Europe, including defragmentation of the existing suid population due to climatic changes during the Early Pleistocene that led to aridification and reduction of forest biotopes. However, increased competition among the members of the omnivore guild (i.e., suids, ursids and hominins) provides a complementary, rather than alternative, explanation for suid extinction, shedding light on a new scenario of increased competition among the members of this guild.

Unlike the suids, the omnivore bear *U. etruscus* was clearly not specialized in nut-cracking, as evidenced in this paper by tooth microwear analysis. In fact, ursids do not rely on these resources in winter, because they hibernate during this season, which allows them to avoid the food shortening of the cold period (although prolonged dormancy was not the cause of elevated δ^13^N values of *U. etruscus* in Venta Micena, see above). In addition, bears are not bone crackers and their scavenging behavior relates exclusively to flesh consumption in the case of large ungulate carcasses. In contrast, they can be opportunistic predators of small-to-medium sized ungulates and, specially, as was suggested at Venta Micena based on biogeochemistry, both *U. etruscus* and the extant *U. arctos* can feed on fish^51^, and also on soft fruits during the warm period of the year.

Our hypothesis tentatively suggests how two different taxa -*U. etruscus* and *Homo*- that are well represented by skeletal remains at Dmanisi and that occupied an ecological niche in which their feeding resources partially overlapped, could coexist during the Early Pleistocene across Eurasia without ecological exclusion of each other. In contrast, the nearly absence of suids at Dmanisi, where they are only recorded by an isolated premolar, suggests that their coexistence with the hominins and bears was more difficult, which probably accelerated the extinction of S*us strozzii*.

## Materials and Methods

### Data

The fossil specimens from Dmanisi are housed in the S. Janashia Museum, National Museums of Georgia (Tbilisi). The ursid collection is mainly composed of craniodental specimens, including 90 specimens represented by teeth and 14 mandibles and hemi-mandibular fragments (see Supplementary Data). We have studied metrically ca. 100 dental specimens and mandibles.

### Systematic paleontology

All the dentognathic ursid fossil material from Dmanisi was measured and described (Table S2). Measurements were taken with a digital caliper (0.05 mm approx. error). We used published and unpublished data (from J.M.-M. and B.F.) of different fossil ursids preserved at different Plio-Pleistocene localities from Europe for comparative purposes: (1) *U. etruscus* from Venta Micena, Fuente Nueva 3, Barranco León^31^ (Spain), Upper Valdarno^42^, Olivola, Pietrafita and Pirro Nord (Italy) and Saint-Vallier^41,43^ (France); (2) *Ursus deningeri* from Trinchera Dolina^76^ (Spain), Untermassfeld^77^ (Germany), Aragó^78^,Vallonnet^79^, Château and Azé^80^ (France)and Stránská Skála^81^ (Czeck republic).

### Quantifying variation among teeth

In order to test if the dentognatic differences among the ursid specimens preserved at Dmanisi could reflect intrapopulation variability resulting from sexual dimorphism, a comparative analysis with a number of species and subspecies of living bears was performed. Specifically, data on the maximum mesiodistal length and buccolingual breadth of the upper and lower canines, fourth premolars and molars of all extant ursids were collected. We sampled only adult individuals –i.e., with complete tooth eruption of permanent dentition, and we made efforts to collect similar numbers of males and females, although this ultimately depended on availability in museum collections. The numbers of males/females per species is described in Table 1 (see results) and the specimens were collected at the American Museum of Natural History (AMNH), the Museum für Naturkunde (MFN), and the Natural History Museum of London (NHM). The degree to which the differences among the tooth specimens measured from Dmanisi could reflect intrapopulation variability resulting from sexual dimorphism was investigated by analyzing the values of the coefficient of variation (CV) for these teeth in populations of modern bear species. In these species, male and female skulls were sampled randomly. The only species excluded from this analysis was the South American Andean bear (*Tremarctos ornatus*), because it is represented in our database by only three specimens. For one species, the brown bear (*U. arctos*), several populations were analyzed and the CV values obtained separately in them were compared with the mean for the species, which allowed to test for interpopulation differences in this species. The CV values estimated in the extant bear species were then compared to those obtained for populations of *U. etruscus* and *U. deningeri* from several Early and Middle Pleistocene sites, including Dmanisi, and also to the pooled CV values of both extinct species.

### Multivariate morphometrics

The possible morphological resemblance of *U. etruscus* and *U. deningeri* with the living bears were explored by Principal Components Analyses (PCA) performed with the correlation semimatrices for the upper and lower tooth measurements, respectively (see Supplementary Information for details). Moreover, the feeding adaptations on the population of *U. etruscus* from Dmanisi was explored by Canonical Variates Analyses (CVA), in order to find those features of the upper and lower dentition that best distinguish among basic feeding types in the living bears. Following the classification of Figueirido^37^, each modern species was grouped in one of the following three dietary groups: (i) herbivores, a category that includes only one species, the giant panda (*Ailuropoda melanoeluca*); (ii) omnivores, a feeding type represented by the brown bear (*U. arctos*), the American black bear (*Ursus americanus*), the Asiatic black bear (*Ursus tibethanus*) and the South American Andean bear (*Tremarctos ornatus*); and (iii) insectivores-carnivores, including the Malayan Sun bear (*Helarctos malayanus*), the sloth bear (*Ursus ursinus*) and the polar bear (*Ursus maritimus*). It is worth noting that following van Heteren^38^, *H. malayanus* was considered in this study as an insectivore and *T. ornatus* as an omnivore. The canonical functions derived for discriminating among herbivores, omnivores and insectivores-carnivores were obtained using the direct method for inclusion of variables in the canonical functions. The percentages of specimens of the living species correctly classified to one dietary group were assessed with the leave-one-out cross-validation procedure using SPSS Statistics, Version 19.

### Tooth microwear analysis

We used the methodology published by Solounias and Semprebon^82^, but adapted to ursids by Münzel^26^. Thirty-five teeth from Dmanisi were analyzed, including 4 p4s (D355 l., D218 r., D36 r., and D36 l.), 1 P4 (D2215 r.), 9 m1s (D1277 r., D1278 l., D5063 r., D2219 l., D4940 l, D2211 r., D2211 l., D3935 r., and D218 r.), 2 M1s (D50 r., and D2214 l.), 9 m2s (D1277 r., D5063 r., D1029 l., D4940 l., D2219 l., D1394 r., D2211 l., D2211 l., and D 218 r.), 3 M2s (D2533 l., D52 r., and D4713 r.), and 7 m3s (D1029 r., D5063 r., D5355 l., D1277 r., D355 l., D2219 l., and D2211 r.), corresponding to a minimum number of five individuals. We included data of fossil specimens from Venta Micena, Barranco León-D and Fuente-Nueva-3 (n = 6) for comparative purposes^31^. Moreover, we included tooth-microwear data of living bears with well-known diets, including *U. arctos* (n = 17) from Münzel^26^ and *U. maritimus* (n = 11) from Dewar^64^. All fossil specimens were cleaned prior to the casting process with cotton swabs soaked with acetone, and later with ethanol. This procedure is aimed to remove any dust and other chemicals left on the surface during the restoration process (e.g., paraloid, a thermoplastic resin). Impressions were obtained using polyvinyl-siloxane silicone (Heraeus Provil novo). Positive casts were made using epoxy resin (Epoxy-150 and K-151). The specimens were checked under a Zeiss Stemi 2000C stereomicroscope with a magnification of 35×. Microwear analysis was focused on two representative areas of the tooth, the grinding facet (occlusal surface) and the slicing facet (the surface of the crown on the lingual or buccal sides). Carnassial teeth (i.e., upper P4 and lower m1) use to be the most analyzed teeth in microwear studies of carnivores (e.g., Goillot *et al*. 2009). However, due to expanded talonid of the m1 in ursids, the carnassial blade is relatively functionally insignificant^83^ and therefore microwear data can only be collected from its talonid cusps surface. We used a standard 0.16 mm^2^ ocular reticle to quantify the number of small pits (very regular with sharp, circular and distinct borders, and very refractive or shiny) and large pits (deeper, less refractive and at least about twice the diameter of small pits), scratches (elongated scars with parallel sides) and gouges (large features with irregular borders). Puncture pits are very deep, symmetrical and with regular margins. Cross scratches are those scratches oriented somewhat perpendicularly to the majority of scratches observed on the dental enamel. The Scratch Width Score (SWS) represents the presence of fine scratches (SWS = 0), the mix of fine and coarse scratches (SWS = 1) and the presence of coarse scratches (SWS = 2). Gray scale high-resolution microphotographs were prepared with a 0.1 mm scale. We analyzed the number of scratches (NS), the number of pits (NP), the number of cross scratches (XS), the number of puncture pits (Npp), the number of gouges (G) and the number of hypercoarse scratches (Nhyper_cs) with PCA (Principal Component Analysis) using the semimatrix of correlations among these variables in order to assess for sample variability and to compare microwear data among extinct and extant ursids. All variables were log-transformed prior to analysis for scaling the microwear variables. Casting and analytical processes were conducted by the same person (TM) to avoid any inter-preparer and observer bias.

## Supplementary information

Supplementary Information

## Data Availability

All data generated or analysed during this study are included in this published article (and its Supplementary Information Files).
