# Author Correction: The bears from Dmanisi and the first dispersal of early *Homo* out of Africa

**DOI:** 10.1038/s41598-021-82920-y

**Published:** 2021-02-03

**Authors:** Tsegai Medin, Bienvenido Martínez-Navarro, Joan Madurell-Malapeira, Borja Figueirido, Giorgi Kopaliani, Florent Rivals, Gocha Kiladze, Paul Palmqvist, David Lordkipanidze

**Affiliations:** 1 IPHES, Institut Català de Paleoecologia Humana i Evolució Social, Zona Educacional, 4, Campus Sescelades URV (Edifici W3), 43007 Tarragona, Spain; 2 Commission of Culture and Sports, Po.Box. 1500, Asmara, Eritrea; 3 ICREA, Pg. Lluís Companys 23, 08010 Barcelona, Spain; 4 Àrea de Prehistòria, Universitat Rovira i Virgili (URV), Avda. Catalunya 35, 43002 Tarragona, Spain; 5 Institut Català de Paleontologia Miquel Crusafont, Universitat Autònoma de Barcelona, Edifici ICTA-ICP, C/de les columnes s/n Campus de la UAB, Cerdanyola del Vallès, 08193 Barcelona, Spain; 6 Departamento de Ecología y Geología, Facultad de Ciencias, Universidad de Málaga, Campus de Teatinos, 29071 Málaga, Spain; 7 National Museum of Georgia, 0105 Tbilisi, Georgia; 8 Tbilisi State University, Tbilisi, Georgia

Correction to: *Scientific Reports* https://doi.org/10.1038/s41598-019-54138-6, published online 28 November 2019

This Article contained errors.

In the original Acknowledgements Dr P. Mazza was incorrectly listed. This has now been fixed in the PDF and HTML versions of the Article.

