# Supplementary material for: The bears from Dmanisi and the first dispersal of early Homo out of Africa
Source: Sci Rep. 2019 Nov 28;9:17752. doi: 10.1038/s41598-019-54138-6 (PMC6882906; doi:10.1038/s41598-019-54138-6)
Supplement: Supplementary file 1 — Supplementary Information [file 41598_2019_54138_MOESM1_ESM.docx]

**Supplementary information for:**

**The bears from Dmanisi and the first dispersal of early *Homo* out of Africa**

Tsegai Medin^1, 2^, Bienvenido Martínez-Navarro^1,3,4*^, Joan Madurell-Malapeira^5^, Borja Figueirido^6^, Giorgi Kopaliani^7^, Florent Rivals^1,3,4^, Gocha Kiladze^7^, Paul Palmqvist^6^, David Lordkipanidze^7^

^1^ IPHES, Institut Català de Paleoecologia Humana i Evolució Social, Zona Educacional, 4, Campus Sescelades URV (Edifici W3), 43007 Tarragona, Spain

^2^ Commission of Culture and Sports (Eritrea), Po.Box. 1500, Asmara^3^ ICREA, Pg. Lluís Companys 23, 08010 Barcelona, Spain

^4^ Àrea de Prehistòria, Universitat Rovira i Virgili (URV), Avda. Catalunya 35, 43002 Tarragona, Spain

^5^ Institut Català de Paleontologia Miquel Crusafont, Universitat Autònoma de Barcelona, Edifici ICTA-ICP, C/ de les columnes s/n Campus de la UAB, Cerdanyola del Vallès, 08193 Barcelona, Spain

^6^ Departamento de Ecología y Geología, Facultad de Ciencias, Universidad de Málaga, Campus de Teatinos, 29071 – Málaga, Spain

^7^ National Museum of Georgia, 0105 Tbilisi, Georgia

^*^ Corresponding author

**Supplementary background**

**The archaeopaleontological record of** **Dmanisi**

The temporal and geographic setting of Dmanisi, the preservational completeness of its faunal and hominin record, and the huge record of Oldowan tools, all make this site crucial for understanding human evolution at the border of Europe during late Early Pleistocene times. The large mammal fauna identified at the site includes: *Canis etruscus, Vulpes alopecoides, Ursus etruscus, Martes* sp., *Meles* sp., *Pliocrocuta perrieri, Pachycrocuta* sp., *Lynx issiodorensis, Acinonyx pardinensis, Panthera onca* ssp. (= *gombaszoegensis*), *Megantereon whitei*, *Homotherium latidens,* *Mammuthus meridionalis,* *Equus stenonis, Equus* aff. *altidens*, *Stephanorhinus etruscus,* *Pseudodama nestii, Praemegaceros obscurus, Arvernoceros insolitus, Cervalces gallicus, Palaeotragus priasovicus, Bison (Eobison) georgicus, Gallogoral meneghinii sickenbergii, Capra dalii, Soergelia* cf. *minor, Praeovibos sp., Pontoceros surprine,* and Antilopini indet.^4,15,48,49,87,88,89,90,91,^*.* The identified small mammal fauna includes: *Beremendia fissidens, Sorex* sp., cf. *Ochotona lagreli,* cf. *Hypolagus brachygnathus*, *Apodemus* aff. *atavus, Cricetulus* sp., *Tcharinomys tornensis, Mimomys pliocaenicus, Parameriones* aff. *obeidiensis, Hystrix* *refossa*^4,92^. Fossil herpetofaunal remains includes: *Bufo ex. gr. viridis, Testudo graeca, Lacerta* ex. gr. *viridis, Elaphe* ex. gr. *quatuorlineata, Natrix* sp.*,* and Colubridae indet.^50^.

Research work over three decades at the site has yielded around 40 hominin remains, including 5 mostly complete skulls, a paleodeme that represents the earliest hominin record out of Africa^1,4,91,93,94,95^. The stone tool assemblage from Dmanisi is a core and flake industry similar to the Oldowan chopping-tool industry of East Africa^4,11,91,96^.

Paleontological and palynological remains suggest that Dmanisi was a mixed woodland environment during the late Early Pleistocene, with a slightly warmer and drier climate compared to the extant Mediterranean climate in this region. Recently, Blain^50^ estimated a mean annual temperature (MAT) of 13.1 ± 2.4 ºC and a mean annual mean precipitation (MAP) of 635 ± 191mm for the Dmanisi region during the Early Pleistocene. The finding of palynological and siliceous phytoliths is interpreted as an indication of increasing aridity during the Dmanisi deposits, with a considerable reduction of wooded areas and the spreading of plain vegetation^46,97,98^.

**Supplementary Materials and Methods**

**Referred fossil specimens of ursids from Dmanisi:**

*Upper dentition*: D1190-Left C; D1823-Right C; D1825-Right M2; D1831-Right M2; D2124-Left C; D2516-Left M2; D2517-Left C; D2325-Right C; D (s/n)-Left C; D626-Left P4; D4473-Left M1; D-683-Left M1; D49-Left M2; D50-Right M1; D52-Right M2; D55-Right P4; D4713-Left M2; D2185-Left M2; D2533-Left M2; D2214-Right palate fragment with P4-M1; D2215-Right P4; D300-Palate with left I1-P1 and P3-M2, and right I1-C and P2-P4; D1725-Palate fragment with right C-P2 and left P1-P2; D2213-Premaxilla with right and left I1-I3; D622-621-Right palate fragment with P4-M2; D809- Palate fragment with left C-P1, alveolus P2, and P3-M2, and right P4-M2.

*Lower dentition:* D5355-Left m3; D1277-Right corpus with p4-m3 in which i1-c are preserved in anatomical connection to the right corpus (p1, p2 and p3 are not preserved); D1278-Left corpus with i2-i3, c, p1, broken p3, and p4-m3; D5063-Right corpus with canine and m1-m3, and alveoli of i2, i3, p2, p3 and p4; D1029-Right corpus with m2-m3 and alveoli of m1; D2219-Left corpus fragment with m1-m3; D355-Complete mandible with left and right corpus including i2, i3, c, and p4-m3 in each side, and alveoli of i1’s, p2’s and p3’s; D4940-Left corpus with c, p4, m1, m2 and alveoli of p2, p3, and m3; D36-Incomplete mandible, with left i1-i3, c, p1,p2, p4, m1-m3, and alveoli of p3, and right i2, i3, c, p4, m1-m3, and alveoli of i1,p1, p2, p3; D2211-Mandible with left i1-i3, c, p1, p2, p4, and alveolus of p3, and right i1-i3, c, p1, p3, p4, m1-m3; D3935-Right m1; D1253-Right m2; D1394-Left hemi-mandibular corpus with m2, broken c and alveoli of i3, p1, p2, p3, m1 and m3; D4705-Right c: D624-Left c; D2123-Left c; D1020-Right hemimandible with m1-m3; D218- Right hemimandible with alveolus of i1, i2, i3, canine root, alveolus of p1, p2, p3, and P4-M2; D1848-Right m2; D2573-Right m2; D3935-Left m1; D2584-Right m1.

**Supplementary Figures**

**
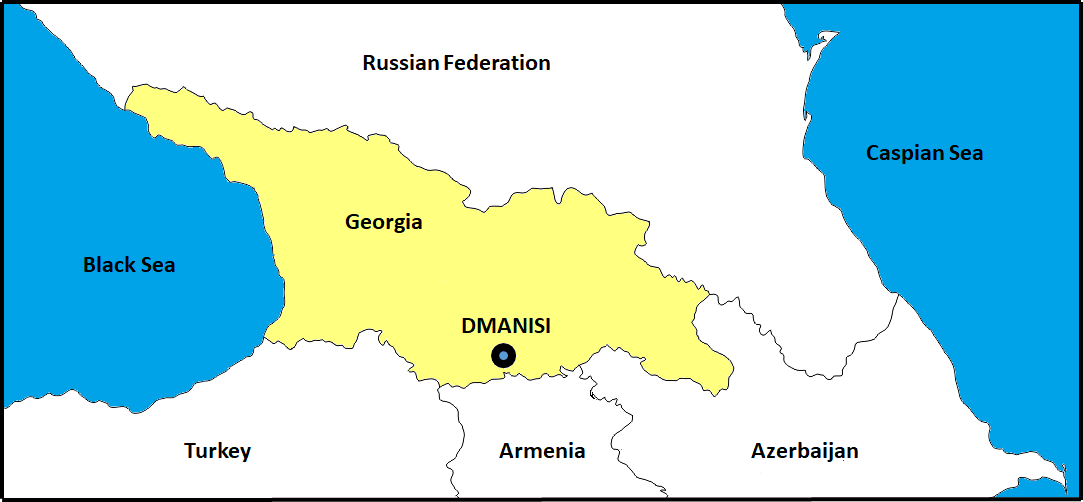
**

**Supplementary Figure 1.** The geographical location of the paleoanthropological site of Dmanisi, Georgia.

**
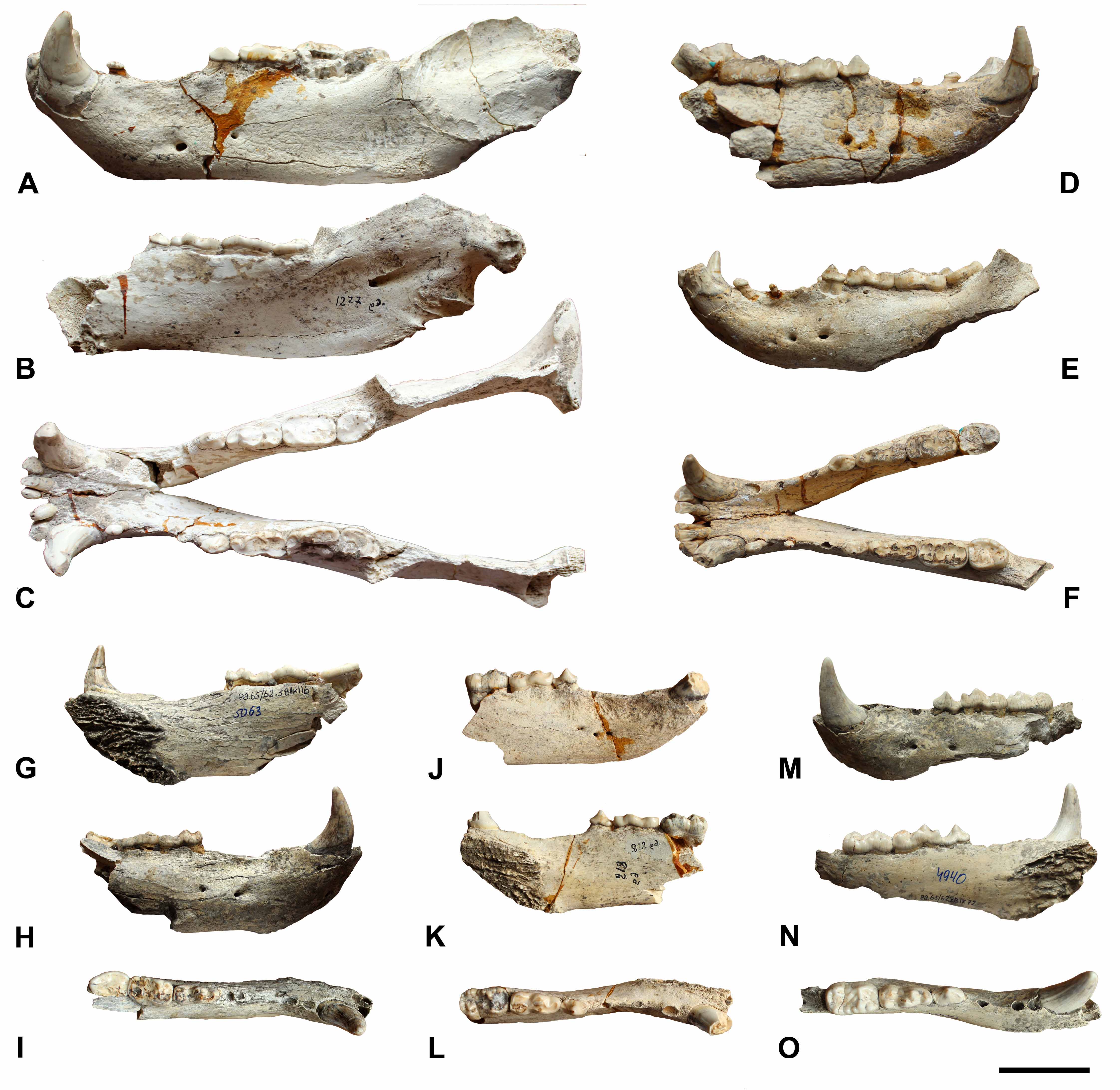
**

**Supplementary Figure 2.** *Ursus etruscus* mandibles. D1277-1278 in (A) buccal view, (B) lingual view, (C) occlusal view (published in Vekua, 1995: plate 14). D218 in (D) right buccal view, (E) left buccal view (published in Vekua, 1995: plate 17), (F) occlusal view (published in Vekua, 1995: plate 16). D5063 in (G) lingual view, (H) buccal view, (I) occlusal view. D218 in (J) buccal view, (K) lingual view, (L) occlusal view. D4940 in (M) buccal view, (N) lingual view, (O) occlusal view. Scale bar =5 cm.

**
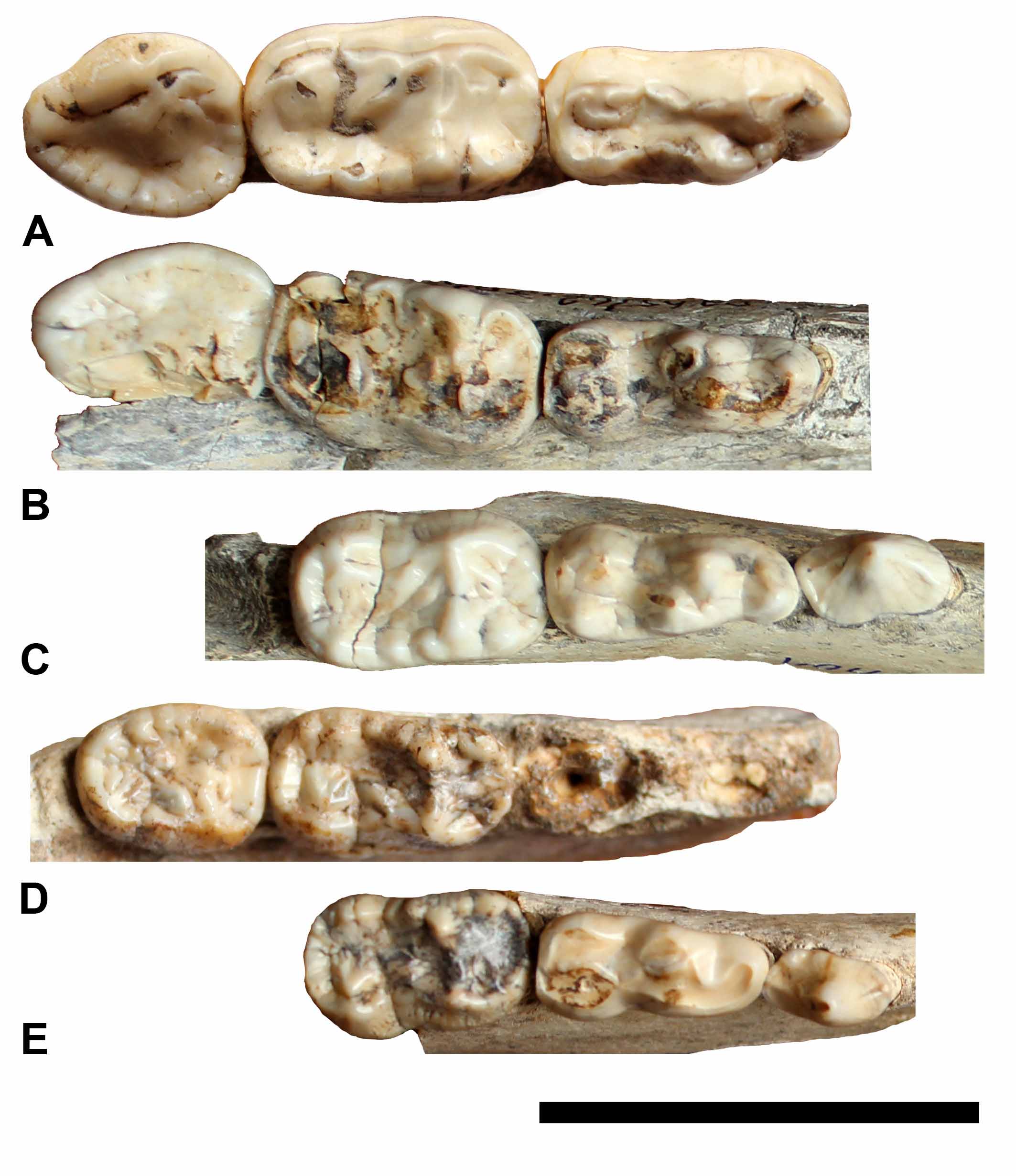
**

**Supplementary Figure 3.** A comparison of *Ursus etrsucus* lower dentition from Dmanisi in occlusal view. (A) D2219; (B) D5063; (C) D4940; (D) D1029; (E) D218. Scale bar =5 cm.

**
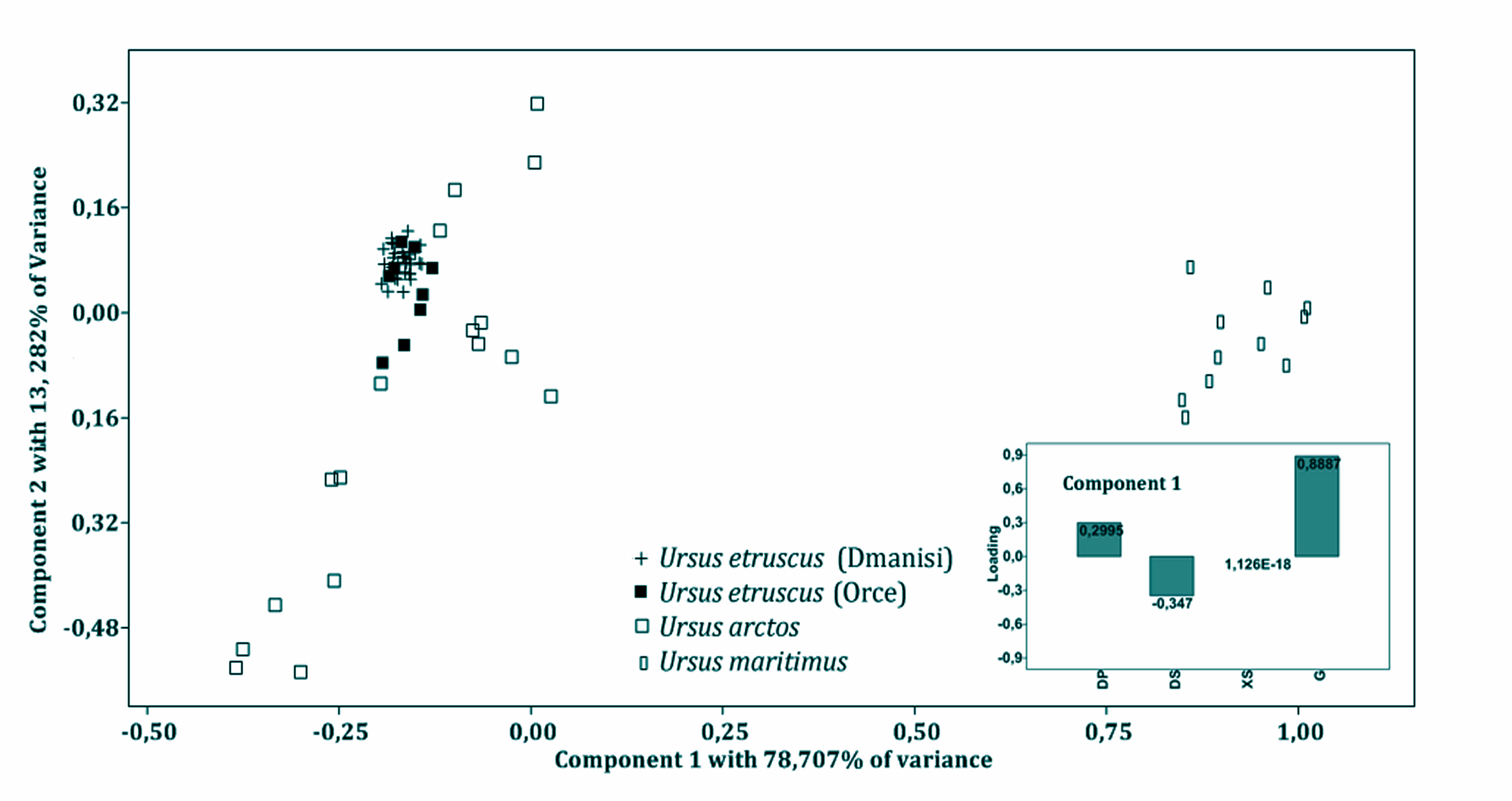
**

**Supplementary Figure 4.** A PCA tooth microwear graph of density of pits (DP), density of scratches (DS), cross scratches (XS) and gouges (G) *Ursus etruscus* (from Dmanisi and Orce), *U. arctos* and *U. maritimus*.

**Supplementary tables**

**Table S1**

| **Site** | **Species** | ***Homo*** | **Chronology** | **Data** |
| --- | --- | --- | --- | --- |
| Dmanisi, Georgia | *U. etruscus* | x | 1.7–1.9 Ma | ^15^ |
| Ubeidiya, Israel | *U. etruscus* | x | ~ 1.4 Ma | ^36,37^ |
| Pirro Nord, Italy | *U. etruscus* | x | 1.3–1.6 Ma | ^87^ |
| Upper Valdarno, Italy | *U. etruscus* | - | 1.77 Ma | ^35^ |
| Venta Micena, Spain | *U. etruscus* | - | 1.5-1.6 Ma | ^31^ |
| Fuente Nueva-3, Spain | *U. etruscus* | x | ~ 1.3 Ma | ^31^ |
| Barranco León, Spain | *U. etruscus* | x | ~ 1.4 Ma | ^31^ |
| St. Vallier, France | *U. etruscus* | - | ~2.2 Ma | ^27^ |
| Senèze, France | *U. etruscus* | - | 2.2 -1.5 Ma | ^28^ |
| Tegelen, The Netherlands | *U. etruscus* | - | 2.4-1.7 Ma | ^32^ |
| Kuruksay, Tadjikistan | *U. etruscus* | - | 1.77–1.95 Ma | ^33^ |

**Supplementary Table 1**. Evidence of *U. etruscus* species during the Early Pleistocene, from main villafranchian sites of Europe, Central Asia, and the Levant. X- shows presence of *Homo.*

**Table S2**

| **Spm. No** | **Spm. typ** | **L** | **W** | **H** | **L.trigonid** | **L.Talonid** | **B.trigonid** | | **B.Talonid** | **L.Int. Lob** | **L.Post. lob** | **B.int. Lob** | **B.post**  **. Lob** |
| --- | --- | --- | --- | --- | --- | --- | --- | --- | --- | --- | --- | --- | --- |
| D300 | L I1 | 5.59 | 7.55 |  |  |  |  |  | |  |  |  |  |
| D300 | R I1 | 5.24 | 7.93 |  |  |  |  |  | |  |  |  |  |
| D2213 | L I1 | 5.78 | 6.43 |  |  |  |  |  | |  |  |  |  |
| D2213 | R I1 | 6.96 | 7.50 |  |  |  |  |  | |  |  |  |  |
| D1278 | L i2 | 9.27 | 5.75 |  |  |  |  |  | |  |  |  |  |
| D300 | L I2 | 7.24 | 8.84 |  |  |  |  |  | |  |  |  |  |
| D300 | R I2 | 6.51 | 9.66 |  |  |  |  |  | |  |  |  |  |
| D2213 | L I2 | 7.34 | 8.97 |  |  |  |  |  | |  |  |  |  |
| D2213 | R I2 | 7.28 |  |  |  |  |  |  | |  |  |  |  |
| D1278 | L i3 | 10.63 | 7.75 |  |  |  |  |  | |  |  |  |  |
| D1277 | R i3 | 10.44 | 7.69 |  |  |  |  |  | |  |  |  |  |
| D1277 | R i2 | 9.22 | 5.21 |  |  |  |  |  | |  |  |  |  |
| D36 | L i1 | 5.70 | 4.20 |  |  |  |  |  | |  |  |  |  |
| D36 | L i2 | 7.91 | 6.08 |  |  |  |  |  | |  |  |  |  |
| D36 | L i3 | 8.96 | 8.27 |  |  |  |  |  | |  |  |  |  |
| D36 | R i2 | 7.60 | 5.40 |  |  |  |  |  | |  |  |  |  |
| D36 | R i3 | 8.60 | 8.73 |  |  |  |  |  | |  |  |  |  |
| D355 | R i2 | 9.24 | 5.66 |  |  |  |  |  | |  |  |  |  |
| D355 | L i3 | 8.85 | 8.38 |  |  |  |  |  | |  |  |  |  |
| D355 | R i3 | 8.85 | 8.38 |  |  |  |  |  | |  |  |  |  |
| D1277 | R i1 | 7.38 | 4.48 |  |  |  |  |  | |  |  |  |  |
| D355 | L i2 | 9.34 | 5.66 |  |  |  |  |  | |  |  |  |  |
| D300 | L I3 | 11.68 | 11.44 |  |  |  |  |  | |  |  |  |  |
| D300 | R I3 | 13.22 | 11.31 |  |  |  |  |  | |  |  |  |  |
| D2213 | L I3 | 7.37 | 9.22 |  |  |  |  |  | |  |  |  |  |
| D2213 | R I3 | 8.42 |  |  |  |  |  |  | |  |  |  |  |
| D1277 | R c | 17.91 | 15.38 |  |  |  |  |  | |  |  |  |  |
| D1278 | L c | 17.53 | 15.55 |  |  |  |  |  | |  |  |  |  |
| D5063 | R c | 17.41 | 13.39 |  |  |  |  |  | |  |  |  |  |
| D4940 | L c | 18.16 | 13.91 |  |  |  |  |  | |  |  |  |  |
| D2325 | R C | 16.37 | 13.81 |  |  |  |  |  | |  |  |  |  |
| D s/n | L C | 14.20 | 11.01 |  |  |  |  |  | |  |  |  |  |
| D4705 | R c | 16.99 | 14.66 |  |  |  |  |  | |  |  |  |  |
| D1190 | L C | 16.16 | 13.22 |  |  |  |  |  | |  |  |  |  |
| D1823 | R C | 14.98 | 11.22 |  |  |  |  |  | |  |  |  |  |
| D2124 | L C | 15.96 | 11.41 |  |  |  |  |  | |  |  |  |  |
| D2517 | L C | 16.45 | 12.10 |  |  |  |  |  | |  |  |  |  |
| D300 | L C | 27.57 | 17.85 |  |  |  |  |  | |  |  |  |  |
| D300 | R C | 16.11 | 18.95 |  |  |  |  |  | |  |  |  |  |
| D1725 | L C | 21.92 | 13.54 |  |  |  |  |  | |  |  |  |  |
| D1725 | R C | 19.64 | 12.67 |  |  |  |  |  | |  |  |  |  |
| D809 | R C | 19.12 | 13.18 |  |  |  |  |  | |  |  |  |  |
| D624 | L c | 14.55 | 10.63 |  |  |  |  |  | |  |  |  |  |
| D2123 | L c | 13.83 | 10.56 |  |  |  |  |  | |  |  |  |  |
| D2211 | R c | 12.68 | 10.38 |  |  |  |  |  | |  |  |  |  |
| D2211 | L c | 12.67 | 10.41 |  |  |  |  |  | |  |  |  |  |
| D355 | L c | 12.67 | 11.28 |  |  |  |  |  | |  |  |  |  |
| D355 | R c | 12.68 | 11.29 |  |  |  |  |  | |  |  |  |  |
| D36 | L c | 12.25 | 11.56 |  |  |  |  |  | |  |  |  |  |
| D36 | R c | 12.38 | 11.36 |  |  |  |  |  | |  |  |  |  |
| D218 | R c | 18.55 | 13.22 |  |  |  |  |  | |  |  |  |  |
| D1278 | L p1 | 8.51 | 6.04 |  |  |  |  |  | |  |  |  |  |
| D36 | L p1 | 8.54 | 5.69 |  |  |  |  |  | |  |  |  |  |
| D2211 | L p1 | 7.70 | 4.79 |  |  |  |  |  | |  |  |  |  |
| D300 | L P1 | 7.06 | 5.63 |  |  |  |  |  | |  |  |  |  |
| D1725 | L P1 | 6.39 | 4.59 |  |  |  |  |  | |  |  |  |  |
| D1725 | R P1 | 7.40 | 5.80 |  |  |  |  |  | |  |  |  |  |
| D809 | L P1 | 7.43 | 4.73 |  |  |  |  |  | |  |  |  |  |
| D36 | L p2 | 5.05 | 3.15 |  |  |  |  |  | |  |  |  |  |
| D2211 | L p2 | 4.49 | 4.13 |  |  |  |  |  | |  |  |  |  |
| D300 | R P2 | 4.54 | 3.95 |  |  |  |  |  | |  |  |  |  |
| D1725 | L P2 | 5.62 | 4.11 |  |  |  |  |  | |  |  |  |  |
| D1725 | R P2 | 5.78 | 4.55 |  |  |  |  |  | |  |  |  |  |
| D300 | L P3 | 7.05 | 5.51 |  |  |  |  |  | |  |  |  |  |
| D300 | R P3 | 6.69 | 5.40 |  |  |  |  |  | |  |  |  |  |
| D809 | L P3 | 6.94 | 4.53 |  |  |  |  |  | |  |  |  |  |
| D1277 | R p4 | 13.18 | 8.31 | 5.80 |  |  |  |  | |  |  |  |  |
| D1278 | L p4 | 14.61 | 8.74 | 6.98 |  |  |  |  | |  |  |  |  |
| D355 | L p4 | 13.21 | 8.10 | 7.94 |  |  |  |  | |  |  |  |  |
| D355 | R p4 | 13.64 | 8.68 | 8.44 |  |  |  |  | |  |  |  |  |
| D4940 | L p4 | 15.97 | 9.53 | 10.57 |  |  |  |  | |  |  |  |  |
| D36 | L p4 | 12.82 | 8.29 | 8.93 |  |  |  |  | |  |  |  |  |
| D36 | R p4 | 12.78 | 8.37 | 8.64 |  |  |  |  | |  |  |  |  |
| D2211 | L p4 | 12.22 | 7.68 | 9.56 |  |  |  |  | |  |  |  |  |
| D218 | R p4 | 13.35 | 6.94 |  |  |  |  |  | |  |  |  |  |
| D2211 | R p4 | 11.65 | 7.80 | 9.71 |  |  |  |  | |  |  |  |  |
| D55 | R P4 | 16.43 | 12.09 | 10.21 |  |  |  |  | |  |  |  |  |
| D2215 | L P4 | 17.04 | 11.27 | 11.77 |  |  |  |  | |  |  |  |  |
| D2214 | L P4 | 15.82 | 11.18 |  |  |  |  |  | |  |  |  |  |
| D300 | L P4 | 18.44 | 12.83 |  |  |  |  |  | |  |  |  |  |
| D300 | R P4 | 18.54 | 12.24 |  |  |  |  |  | |  |  |  |  |
| D626 | L P4 | 15.96 | 11.42 |  |  |  |  |  | |  |  |  |  |
| D622-621 | R P4 | 15.74 | 11.28 |  |  |  |  |  | |  |  |  |  |
| D809 | L P4 | 17.31 | 10.08 |  |  |  |  |  | |  |  |  |  |
| D809 | R P4 | 17.45 | 11.02 |  |  |  |  |  | |  |  |  |  |
| D1277 | R m1 | 28.10 | 13.37 | 7.10 | 17.39 | 10.77 | 11.02 | 13.60 | | 16.32 | 8.41 | 9.17 | 10.67 |
| D1278 | L m1 | 28.24 | 13.68 | 6.85* | 19.23 | 9.96 | 11.80 | 13.93 | | 16.02 | 8.62 | 8.91 | 9.66 |
| D5063 | R m1 | 26.63 | 11.41 | 10.26 | 16.32 | 8.84 | 9.58 | 11.40 | | 12.87 | 7.19 | 5.36 | 9.22 |
| D2219 | Lm1 | 24.47 | 10.48 | 11.55 | 16.57 | 7.21 | 10.15 | 10.90 | | 14.97 | 7.41 | 4.55 | 5.88 |
| D355 | L m1 | 26.25 | 13.45 | 8.21* |  |  |  |  | |  |  |  |  |
| D355 | R m1 | 26.16 | 13.42 | 8.14* |  |  |  |  | |  |  |  |  |
| D4940 | L m1 | 25.81 | 11.46 | 10.99 | 16.03 | 8.85 | 9.25 | 11.44 | | 12.72 | 9.13 | 5.13 | 6.50 |
| D36 | L m1 | 23.94 | 10.32 | 10.15 | 15.88 | 7.98 | 9.28 | 9.77 | | 13.03 | 7.47 | 5.68 | 6.70 |
| D36 | R m1 | 24.08 | 10.71 | 10.21 | 15.53 | 7.98 | 9.29 | 9.78 | | 13.03 | 7.78 | 5.21 | 6.69 |
| D3935 | R m1 | 23.58 | 10.59 | 5.65* | 15.11 | 7.42 | 8.04 | 9.54 | | 13.16 | 6.44 | 5.08 | 6.67 |
| D2584 | R m1 |  | 14.14 |  |  |  |  |  | |  |  |  |  |
| D3935 | R m1 | 24.01 | 10.28 |  | 14.63 |  |  |  | |  |  |  |  |
| D218 | R m1 | 23.61 | 11.63 |  | 14.74 |  |  |  | |  |  |  |  |
| D4473 | L M1 | 23.99 | 18.87 | 10.47 | 11.02 | 12.63 | 16.67 | 18.50 | | 8.86 | 12.85 | 7.95 | 9.33 |
| D50 | R M1 | 22.38 | 16.24 | 9.72 | 10.98 | 12.62 | 15.31 | 15.55 | | 8.06 | 10.74 | 7.11 | 9.76 |
| D2214 | L M1 | 23.05 | 16.31 | 11.12 | 10.88 | 12.49 | 15.70 | 16.01 | | 9.61 | 11.48 | 8.32 | 9.57 |
| D683 | L M1 | 22.58 | 16.77 |  |  |  |  |  | |  |  |  |  |
| D300 | L M1 | 24.08 | 18.46 |  |  |  |  |  | |  |  |  |  |
| D300 | R M1 | 23.95 | 18.09 |  |  |  |  |  | |  |  |  |  |
| D622-621 | R M1 | 21.94 | 16.50 |  |  |  |  |  | |  |  |  |  |
| D809 | L M1 | 24.77 | 19.10 |  |  |  |  |  | |  |  |  |  |
| D809 | R M1 | 25.09 | 20.01 |  |  |  |  |  | |  |  |  |  |
| D1277 | R m2 | 26.64 | 17.96 | 6.60* |  |  |  |  | |  |  |  |  |
| D1278 | L m2 | 26.55 | 17.97 |  |  |  |  |  | |  |  |  |  |
| D5063 | Rm2 | 26.78 | 15.75 | 7.86 |  |  |  |  | |  |  |  |  |
| D1029 | R m2 | 23.17 | 13.70 | 8.64 | 14.28 | 8.41 | 12.40 | 12.90 | | 13.51 | 6.85 | 7.98 | 9.12 |
| D2219 | Lm2 | 23.72 | 14.22 | 7.34 | 16.52 | 7.52 | 14.00 | 13.33 | | 15.86 | 6.72 | 7.87 | 8.77 |
| D355 | L m2 | 27.90 | 18.11 |  |  |  |  |  | |  |  |  |  |
| D355 | R m2 | 27.92 | 18.18 |  |  |  |  |  | |  |  |  |  |
| D4940 | L m2 | 27.26 | 14.70 | 11.07 | 15.63 | 9.55 | 14.19 | 14.63 | | 16.12 | 7.51 | 8.49 | 9.36 |
| D36 | L m2 | 25.11 | 15.00 |  | 15.13 | 8.77 | 13.62 | 13.59 | | 15.49 | 7.91 | 9.21 | 9.39 |
| D36 | R m2 | 25.03 | 15.08 |  | 15.28 | 8.87 | 13.48 | 13.82 | | 15.13 | 8.01 | 9.06 | 9.40 |
| D1253 | R m2 | 24.36 | 15.67 | 8.53 | 15.41 | 8.76 | 13.20 | 14.61 | | 13.74 | 6.51 | 7.46 | 9.26 |
| D1394 | R m2 | 26.56 | 15.96 | 7.61 | 16.26 | 9.05 | 14.11 | 14.77 | | 14.51 | 7.59 | 7.63 | 7.63 |
| D1848 | L m2 | 30.65 | 18.35 |  |  |  |  |  | |  |  |  |  |
| D683 | L m2 |  |  |  |  |  |  |  | |  |  |  |  |
| D1020 | R m2 | 23.14 | 14.74 |  |  |  |  |  | |  |  |  |  |
| D218 | R m2 | 23.38 | 14.66 |  |  |  |  |  | |  |  |  |  |
| D2573 | R m2 | 28.50 | 18.03 |  |  |  |  |  | |  |  |  |  |
| D1848 | R m2 | 31.13 | 19.93 |  |  |  |  |  | |  |  |  |  |
| D1825 | R M2 | 34.95 | 18.93 |  |  |  |  |  | |  |  |  |  |
| D1831 | L M2 | 32.39 | 19.17 |  |  |  |  |  | |  |  |  |  |
| D2516 | L M2 | 33.59 | 18.06 |  |  |  |  |  | |  |  |  |  |
| D49 | L M2 | 34.31 | 19.51 |  |  |  |  |  | |  |  |  |  |
| D2185 | L M2 | 24.18 | 19.19 |  |  |  |  |  | |  |  |  |  |
| D2573 | L M2 | 28.29 | 18.10 |  |  |  |  |  | |  |  |  |  |
| D52 | R M2 | 28.23 | 20.43 | 8.81 | 11.92 |  |  | 19.36 | | 12.92 |  | 10.13 |  |
| D4713 | L M2 | 35.83 | 18.56 | 9.31 | 13.23 | 22.86 | 18.07 | 17.38 | | 12.21 | 20.80 | 8.62 | 9.70 |
| D300 | L M2 | 34.02 | 19.09 |  |  |  |  |  | |  |  |  |  |
| D300 | R M2 | 36.27 | 19.13 |  |  |  |  |  | |  |  |  |  |
| D622-621 | R M2 | 33.53 | 18.82 |  |  |  |  |  | |  |  |  |  |
| D809 | L M2 | 40.68 | 21.64 |  |  |  |  |  | |  |  |  |  |
| D809 | R M2 | 39.91 | 21.72 |  |  |  |  |  | |  |  |  |  |
| D5063 | R m3 | 22.53 | 14.81* |  |  |  |  |  | |  |  |  |  |
| D5355 | Lm3 | 20.68 | 16.96 | 7.19 | 16.74 | 5.81 | 16.95 | 10.25 | | 11.78 | 5.18 | 10.44 | 6.05 |
| D1277 | R m3 | 22.41 | 14.71 | 4.11* |  |  |  |  | |  |  |  |  |
| D1278 | L m3 | 20.50 | 15.62 |  |  |  |  |  | |  |  |  |  |
| D1029 | R m3 | 18.42 | 14.02 | 6.24 | 12.07 | 6.97 | 14.51 | 11.53 | | 9.65 | 6.01 | 9.43 | 7.85 |
| D36 | L m3 | 19.13 | 15.29 |  | 14.13 | 7.15 | 14.70 | 10.91 | | 10.19 | 5.79 | 11.16 | 7.75 |
| D36 | R m3 | 19.23 | 15.03 |  |  |  |  |  | |  |  |  |  |
| D355 | L m3 | 18.38 | 17.17 |  |  |  |  |  | |  |  |  |  |
| D355 | R m3 | 18.53 | 17.27 |  |  |  |  |  | |  |  |  |  |
| D1020 | R m3 | 18.29 | 14.56 |  |  |  |  |  | |  |  |  |  |
| D2219 | L m3 | 17.90 | 15.29 | 5.76* | 13.37 | 5.80 | 15.29 | 9.73 | | 11.20 | 3.97 | 10.77 | 6.31 |

**Supplementary Table S2**. Supplementary data material of dental metrics of *Ursus* species from Dmanisi. L (length), W (width), H (height), L.trigonid (Length of trigonid) L.Talonid (Length of talonid) B.trigonid (Breadth of trigonnid) B.Talonid (Breadth of talonid) L.Int. Lob (length of internal lobule) L.Post lob (length of posterior lobule) B.int. Lob (Breadth of interior lobule) and B. post. Lob (Breadth of posterior lobule)*.* (*= *approx.* measurement). All measurements are in mm.

**Supplementary References (continued from main text)**

87. Vekua A. *et al.* New site of the Neogene Vertebrate fauna from Kaspi district. *Bull. Geor. Nat. Acad. Sci.* **6**, 151–157 (2012).

88. Chkchikvadze, V. & Kharabadze E. Amphibians and Reptiles from the Early Pleistocene of Georgia (Abstracts 14^th^ INQUA Congress, Berlin, 1995).

89. Martínez-Navarro, B. & Palmqvist, P. Presence of the African Machairodont *Megantereon whitei* (Broom, 1937) (Felidae, Carnivora, Mammalia) in the Lower Pleistocene site of Venta Micena (Orce, Granada, Spain), with some considerations on the origin, evolution and dispersal of the genus. *J. Archaeol. Sci.* **22**, 569–582 (1995).

90. Gabunia, L., Vekua, A. & Bugianisvili, T.V. Sreda obitanija drevnejsich iskopaemych ljudej Kavkaza. *Izv. Akad. Nauk. Ssr. Ser. A.* **14**, 344–349 (1988).

91. Gabunia, L., Vekua, A. & Lordkipanidze, D. The environmental contexts of early human occupation of Georgia (Transcaucasia). *J. Hum. Evol.* **38**, 785–802 (2000).

92. Furió, M., Agustí, J., Mouskhelishvili, A., Sanisidro, Ó. & Santos-Cubedo, A. The paleobiology of the extinct venomous shrew *Beremendia* (Soricidae, Insectivora, Mammalia) in relation to the geology and paleoenvironment of Dmanisi (Early Pleistocene, Georgia). *J. Vert. Paleontol.* **30**, 928–942 (2010).

93. Gabunia, L., de Lumley, M.A., Vekua, A., Lordkipanidze, D. & Lumley, H. 2002. Découverte d’un nouvel hominidé à Dmanisi*. C.R. Palévol.* **1**, 243–253.

94. Rightmire, G. P., Lordkipanidze, D. & Vekua, A. Anatomical descriptions, comparative studies and evolutionary significance of the hominin skulls from Dmanisi, Republic of Georgia. *J. Hum. Evol.* **50**, 115–41 (2006).

95. Lordkipanidze, D. *et al.* Complete skull from Dmanisi, Georgia, reveals evolutionary biology of early *Homo*. *Science* **342**, 326–331 (2013).

96. Nioradze , M. & Justus, A. Stone tools of the ancient Palaeolithic site Dmanisi In *Dmanisi* (ed. Kopaliani, D.) 140–159 (Metsniereba, 1998).

97. Messager, E. *Apports des études paléobotaniques à la reconstitution paleoenvironnementale du site de Dmanissi et de sa région (Géorgie).* (Unpubl. Ph.D. Dissertation, 2006).

98. Messager E., Lordkipanidze D., Ferring C.R. & Deniaux B. Fossil fruit identification by SEM investigations, a tool for palaeoenvironmental reconstruction of Dmanisi site, Georgia. *J. Archaeol. Sci.* **35**, 2715–2725 (2008).
